# Supplementary material for: Broad-spectrum capture of hundreds of per- and polyfluoroalkyl substances from fluorochemical wastewater
Source: Nat Commun. 2025 Feb 25;16:1972. doi: 10.1038/s41467-025-57272-0 (PMC11861909; doi:10.1038/s41467-025-57272-0)
Supplement: Supplementary file 1 — Supplementary Information [file 41467_2025_57272_MOESM1_ESM.pdf]

Supplementary Information

Broad-spectrum capture of hundreds of per- and polyfluoroalkyl substances in fluorochemical wastewater

Yiyang Liang[1,2#], Lihui Yang[1,2 #], Caiming Tang[1,2#], Ying Yang[1,2], Shangtao Liang[3], Anqi Wang[1,2], Jiale Xu[4], Qingguo Huang[3], Hui Lin[1,2*]

[1] Research Center for Eco-Environmental Engineering, Dongguan University of Technology, Dongguan, 523808, PR China

[2] College of Eco-Environment and Architectural Engineering, Dongguan University of Technology, Dongguan, 523808, PR China

[3] College of Agricultural and Environmental Sciences, Department of Crop and Soil Sciences, University of Georgia, Griffin, GA 30223, United States

[4] Department of Civil, Construction and Environmental Engineering, North Dakota State University, Fargo, North Dakota 58102, United States

[#] Yiyang Liang, Lihui Yang and Caiming Tang contributed equally to this work;

[*]Corresponding author: Hui Lin, Phone: +86-769-22863169, E-mail: linhui@dgut.edu.cn.

37 Pages, 2 Texts, 11 Tables, 21 Figures

Supplementary Text 1 Techno-Economic Analysis (TEA)

The price of PFA694E is \$19.3 per liter, based on an August 2023 quote from Purolite (China) Co., Ltd. for a 5000L batch (excluding shipping). The zinc price is \$3097.1 per ton (September 2023 Chinese market price). The electricity cost is about \$0.1 per kWh for industrial use. The ZnO price is \$3097.1 per ton (September 2023 Chinese market price). The spent PFA694E and Zn hydroxide flocs (assuming 10% water content) with adsorbed PFAS would be incinerated. The cost for hazardous waste incineration treatment is derived from the "Government-Priced Business Service Fee Catalogue (2023 Edition)" and is approximately \$313.6 per ton based on low calorific value incineration. Detailed costs of materials, electricity and incineration etc. are shown in Supplementary Table 12. The cost of the zinc-based EC process (\$ per m^3) was calculated by following equations:

$$\text{Cost}_{\text{EC}}=0.1\times E + \text{Price of Zn} + \text{Treatment Price of Zn hydroxide flocs} \quad \text{(S1)}$$

$$E=\frac{U\times I}{V}\times t_{\text{EC}} \quad \text{(S2)}$$

$$\text{Zinc dosage} = \frac{1000}{V}\times\frac{I\times t_{EC}}{nF}\times M\times\eta \quad \text{(S3)}$$

$$\text{Zn} = \text{Zn dosage}\times V \quad \text{(S4)}$$

$$\text{Zn hydroxide flocs} = \frac{99}{65}\times\frac{1}{0.9}\times\text{Zn} = 1.69\times\text{Zn} \quad \text{(S5)}$$

$$\text{ZnO} = \frac{84}{65}\times\text{Zn} = 1.25\times\text{Zn} \quad \text{(S6)}$$

where E (kWh m^{-3}) is the energy cost of Zn-based EC; and I and t_{EC} refer to the applied current and time during electrocoagulation, respectively; F is the Faraday's constant; n is the number of electrons in $Zn - 2e \rightarrow Zn^{2+}$; M (65 g mol^{-1}) refers the relative molar mass of zinc; η refers the current efficiency of $Zn - 2e \rightarrow Zn^{2+}$, which is determined to 0.91 in the fluorochemical wastewater used in this study. The cost of the PFA694E bed (\$ per m^3) was calculated by following equations:

$$\text{Cost}_{\text{PFA694E bed}} = (\text{Price} + \text{Incineration Treatment Cost})\times\text{PFA694E amount} \quad \text{(S7)}$$

$$\text{PFA694E amount}=\frac{0.625}{9.27\times10^{-7}\text{BV}} \quad \text{(S8)}$$

where BV is the bed volume at the moment of RSSCT breakthrough; 9.27×10^{-7} (m^3) is the volume of PFA694E in the RSSCT column; 0.625 (g) is the mass of PFA694E in the RSSCT column. The total cost (\$ per m^{-3}) of the treatment-train process was calculated by equation S9.

$$\text{Cost}_{\text{Treatment-train}}=\text{Cost}_{\text{EC}}+\text{Cost}_{\text{PFA694E bed}} \quad \text{(S9)}$$

Cost of materials, electricity and waste incineration:

	Unit cost
PFA694E	\$19.3 per L
Zn	\$3097.1 per t
Electricity	\$0.1 per kW h
Hazardous waste	\$313.6 per t

Supplementary Text 2 Life-cycle Environmental Impact Assessment (LCEIA)

Life-cycle assessment is an environmental impact assessment method that considers the material and energy interactions over the life cycle of a product or process that impacts the environment. The functional unit chosen here is 90% or 99% removal of PFAS from 1 m^3 fluorochemical wastewater. In this study, the PFA694E bed operated as a single-use adsorbent, and the spent PFA694E and Zn hydroxide flocs with adsorbed PFAS would be incinerated. The scenarios are modeled as follows: Zn-based EC treatment followed by PFA694E bed treatment (Scenarios 1) and PFA694E bed treatment only (Scenarios 2). An overview of the unit process, materials, and energy considered in the two scenarios is shown as follows:

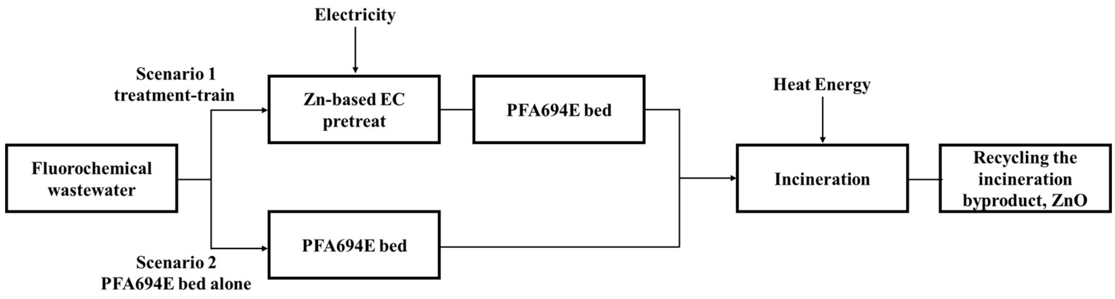

The system diagram includes Zn-based EC treatment process, PFA694E bed sorption process, and the incineration of spent Zn hydroxide flocs/PFA694E. Material and energy inputs to the system include Zn electrode, PFA694E, electric energy to the Zn-based EC process, and heat energy for incineration. All data sources for the unit emission and environmental impact factors were obtained from the database Ecoinvent (version 3.9.1) and European Life Cycle Database (ELCD). In this study, we only consider the carbon footprints (Kg CO_2 eq.).

PFA694E is a new resin with no documented life cycle assessment of its production process, it is assumed that the carbon emissions are substituted by those of a resin with 50% w/w water content, which is equivalent to a resin cross-linked with 6-8% w/w divinylbenzene (DVB) (an 8%

crosslinking rate is assumed in this inventory). Missions for zinc are based on the wet process production of zinc (purity: 99.995%). Carbon emissions for electricity are based on the 2021 national grid average emission factor. The carbon emissions from the incineration process are assumed based on the average value of the Rest of the World (ROW) excluding the European Union and the greenhouse effect of a certain greenhouse gas within 100 years compared to the equivalent effect of carbon dioxide (GWP100). The carbon dioxide emission compensation for the zinc oxide produced is assumed to use the wet process. The carbon-footprint emissions equivalent of each process from the database are shown in Supplementary Table 13. The carbon footprint of the zinc-based EC process (CFP_{EC}, $KgCO_2$ per m^3) was calculated by equation S10.

$$CFP_{EC} = 0.58\times E + 2.7\times Zn + 2.45\times Zn \text{ hydroxide flocs} \quad (S10)$$

The carbon footprint of the PFA694 bed ($CFP_{PFA694E\ bed}$, Kg CO_2 per m^3) was calculated by equation S11.

$$CFP_{PFA694E\ bed} = (3.73+2.45+1.77) \times PFA694E \quad (S11)$$

The carbon footprint of the treatment-train ($CFP_{Treatment\text{-}train}$, Kg CO_2 per m^3) was calculated by equation 12.

$$CFP_{Treatment\text{-}train} = CFP_{EC} + CFP_{PFA694E} \quad (S12)$$

Carbon emissions from materials manufacturing, electricity and waste incineration:

	Carbon equivalent（kg CO_2-eq. per kg）
Zn	2.70
PFA694E	3.73
Electricity	0.58
Hazardous waste treatment	2.45
Incineration of PFA694E*	1.77

*Carbon emissions of PFA694E determined by TOC analyzer

Supplementary Table 1. Information of 107 PFAS in fluorochemical wastewater.

Formula	Monoisotopic molecular weight	MRM transition (m/z)	Concentration (μg L^{-1})	Name	log K_{ow}	Molecular structure	CAS/RN	ChemSpider /PubChem ID
**I. PFCA**								
**Class1 PFCA**								
$C_2HO_2F_3$*	113.99287	113→69	1385.72 ± 5.68	Trifluoroacetic acid (TFA)	0.50	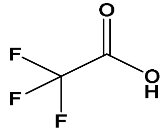	CS:200-929-3 RN:76-05-1	10239201
$C_3HO_2F_5$*	163.98967	163→119	186.23 ± 14.82	Perfluoropropionic acid (PFPrA)	1.47	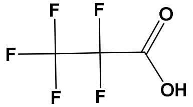	CS:207-021-6 RN:422-64-0	56147
$C_4HO_2F_7$*#	213.98648	213→169	204.75± 15.56	Heptafluorobutyric acid (PFBA)	2.14	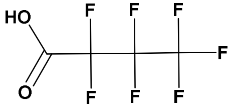	CS:206-786-3 RN:375-22-4	9394
$C_5HO_2F_9$*#	263.98328	263→219	185.61± 17.96	Perfluoropentanoic acid (PFPeA)	2.81	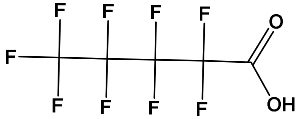	CS:220-300-7 RN:2706-90-3	68426
$C_6HO_2F_{11}$*#	313.98010	313→269	525.20± 4.84	Perfluorohexanoic acid (PFHxA)	3.48	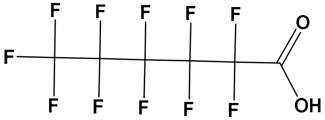	CS:206-196-6 RN:307-24-4	60864
$C_7HO_2F_{13}$*#	363.97690	363→319	426.04 ± 4.66	Perfluoroheptanoic acid (PFHpA)	4.15	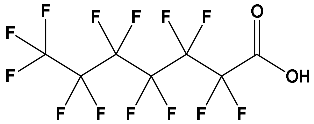	CS:206-798-9 RN:375-85-9	61135
$C_8HO_2F_{15}$*#	413.97369	413→369	23790.00 ± 1393.37	Perfluorooctanoic Acid (PFOA)	4.81	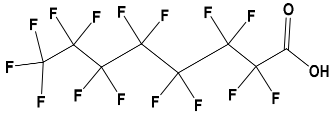	CS:200-659-6 RN:335-67-1	9180
$C_9HO_2F_{17}$*#	463.97052	463→419	21.05 ± 2.04	Perfluorononanoic acid (PFNA)	5.48	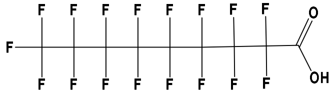	CS:206-801-3 RN:375-95-1	61138

$C_{10}HO_2F_{19}$*	513.96729	513→469	6.32 ± 1.00	Perfluorocapric acid (PFDA)	6.15	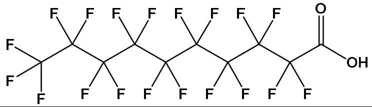	RN:335-76-2	9181
**II. H-PFAA**								
**Class2 H-PFCA or H-PFSA**								
$C_3H_2O_2F_4$*	145.99908	145→101	2590.29 ± 203.85	Flupropanate（H-PFPrA)	0.86	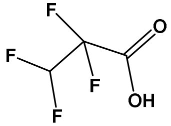	CS:212-049-7<br>RN:756-09-2	62988
$C_4H_2O_2F_6$*$^{\#}$	195.99589	195→151	525.20 ± 4.84	Hexafluorobutanoic acid (H-PFBA)	2.07	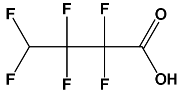	RN:679-12-9	1385589
$C_5H_2O_2F_8$*$^{\#}$	245.99271	245→201	85.20± 6.48	Octafluoropentanoic acid (H-PFPeA)	2.2	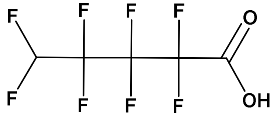	RN:376-72-7	107330
$C_6H_2O_2F_{10}$	295.98950	295→251	182.36 ± 3.92	2,2,3,3,4,4,5,5,6,6-Decafluoro-hexanoic acid (H-PFHxA)	2.87	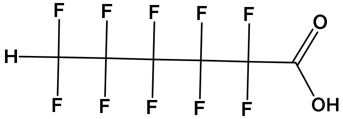	NA	98251620
$C_7H_2O_2F_{12}$*	345.98633	345→301	53.26 ± 2.96	7H-Dodecafluoroheptanoic acid (H-PFHpA)	3.53	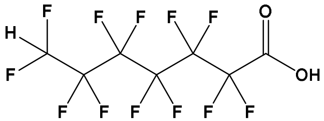	NA	28190785
$C_8H_2O_2F_{14}$*$^{\#}$	395.98312	395→351	136.13 ± 8.96	2,2,3,3,4,4,5,5,6,6,7,7,8,8-Tetradecafluorooctanoic acid (H-PFOA)	4.21	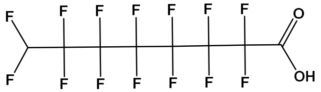	RN:13973-14-3	14172003
$C_9H_2O_2F_{16}$*$^{\#}$	445.97992	445→401	52.76 ± 1.68	9H-Hexadecafluorononanoic acid (H-PFNA)	4.88	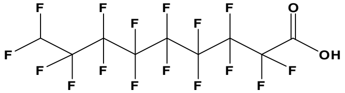	CS:200-944-5<br>RN:76-21-1	6194
$C_{10}H_2O_2F_{18}$*	495.97673	495→451	93.94 ± 3.34	2,2,3,3,4,4,5,5,6,6,7,7,8,8,9,9,10,10-Octadecafluoro-decanoic acid (H-PFDA)	5.54	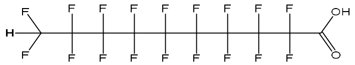	NA	NA
$C_2H_2O_3F_4S$	181.96608	181→101	3.25 ± 0.57	Tetrafluoroethanesulfonic acid (H-TFSA)	0.13	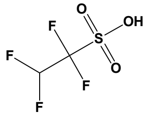	RN:464-14-2	8351896

$C_3H_2O_3F_6S$	231.96289	231→151	1.80 ± 0.11	hexafluoropropanesulfonic acid (H-PFPrSA)	0.84	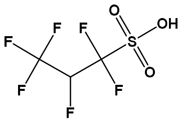	RN:357-31-3	7970395
**Class3 n:(m+1) FTCA**								
$C_4H_5O_2F_3$*	142.02417	141→96	708.35 ± 52.17	2,2,3-Trifluoro-butyric acid (1:3 FTBA395)	1.41	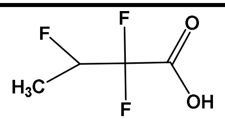	381-97-5 RN:1314967-76-4	32994636
$C_5H_3O_2F_7$*	228.00212	227→182	30.69 ± 2.37	2,2,4,4,5,5,5-Heptafluoropentanoic acid (3:2 FTPeA)	2.93	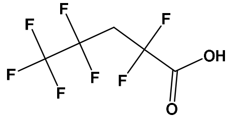	679-46-9 RN:2228328-80-9	65496648
$C_6H_3O_2F_9$	277.99893	277→233	8.24 ± 0.87	2,2,4,4,5,5,6,6,6-Nonafluorohexanoic acid (4:2 FTHxA)	3.59	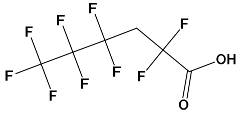	NA	98022553
$C_6H_5O_2F_7$	242.01778	241→196	1.92 ± 0.15	2,2,5,5,6,6,6-Heptafluorohexanoic acid (3:3 FTHxA)	3.42	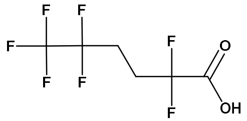	NA	97339106
$C_7H_3O_2F_{11}$	327.99573	327→283	1.29 ± 0.16	3,3,4,4,5,5,6,6,7,7,7-Undecafluoroheptanoic acid (5:2 FTHpA)	3.97	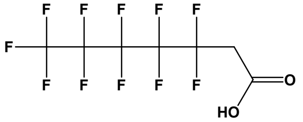	RN:889944-77-8	15880371
$C_8H_3O_2F_{13}$	377.99255	377→333	4.54 ± 0.74	2,2,3,3,4,4,5,5,6,6,7,7,8-Tridecafluoro-octanoic acid (6:2 FTOA)	4.64	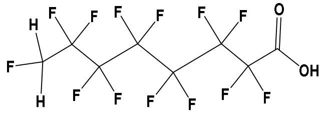	NA	NA
$C_8H_5O_2F_{11}$	342.01138	341→296	3.74 ± 0.62	2,2,3,3,4,4,5,5,6,6,7-Undecafluoro-octanoic acid (5:3 FTOA)	4.38	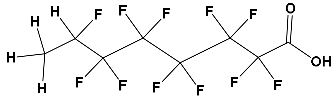	NA	NA
$C_8H_7O_2F_9$*	306.03024	305→260	57.33 ± 2.91	2,2,3,3,4,4,5,5,6-Nonafluoro-octanoic acid (4:4 FTOA)	4.21	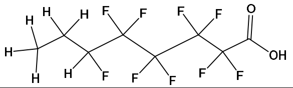	NA	NA
**Class4 n:(m+1) H-FTCA or n:m H-FTSA**								
$C_5H_6O_2F_4$	174.03040	173→128	1.86 ± 0.27	2,2,4,4-Tetrafluoropentanoic acid (2:3 H-FTPeA)	2.51	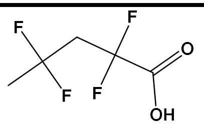	RN:2229223-50-9	65442335

$C_6H_4O_2F_8$*	260.00836	259→214	60.68 ± 2.96	2,2,5,5,5-Pentafluoro-4-(trifluoromethyl)pentanoic acid (4:2 H-FTHxA)	3.28	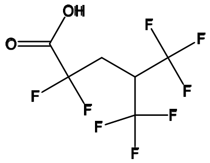	NA	98027550
$C_6H_6O_2F_6$*#	224.02721	223→178	976.50 ± 24.81	3-Difluoromethyl-4,4,5,5-tetrafluoro-pentanoic acid (3:3 H-FTHxA)	2.07	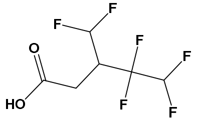	NA	98522519
$C_7H_4O_2F_{10}$*#	310.00516	309→264	111.73 ± 5.65	2,2,3,3,4,4,5,5,6,6-Decafluoroheptanoic acid (5:2 H-FTHpA)	4.03	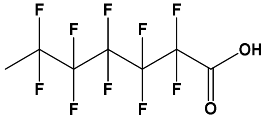	NA	28190785
$C_8H_4O_2F_{12}$	360.00195	359→314	7.89 ± 1.41	2,2,3,3,4,4,5,5,6,6,7,7-Dodecafluoro-octanoic acid (6:2 H-FTOA)	4.70	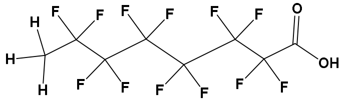	NA	NA
$C_8H_6O_2F_{10}$	324.02081	323→278	9.42 ± 1.54	2,2,3,3,4,4,5,5,6,6-Decafluorooctanoic acid (5:3 H-FTOA)	4.52	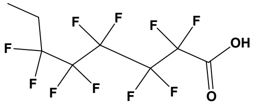	NA	95707742
$C_8H_8O_2F_8$*	288.03964	287→242	73.47 ± 3.13	2,2,3,3,4,4,5,5-Octafluoro-octanoic acid (4:4 H-FTOA)	4.34	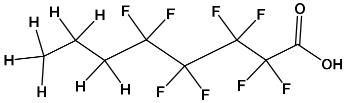	NA	NA
$C_4H_4O_3F_6S$	245.97853	245→165	0.95 ± 0.13	Hexafluoro-2-butanesulfonic acid (3:1 H-FTBSA)	0.84	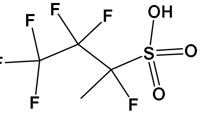	NA	95789274
$C_5H_4O_3F_8S$	295.97534	295→215	1.31 ± 0.13	1,1,2,4,4,5,5,5-Octafluoro-pentane-1-sulfonic acid (4:1 H-FTPeSA)	2.29	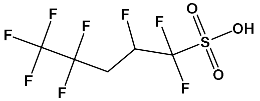	RN:676525-95-4	62982309
$C_7H_6O_3F_{10}S$	359.98779	359→279	1.72 ± 0.50	1,1,2,2,3,3,4,4,5,6-Decafluoro-heptane-1-sulfonic acid (5:2 H-FTHpSA)	3.08	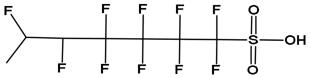	NA	NA
$C_8H_{10}O_3F_8S$	338.02229	337→256	4.65 ± 0.92	1,1,2,2,3,3,4,4-Octafluoro-octane-1-sulfonic acid (4:4 H-FTOSA)	3.84	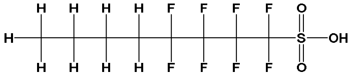	NA	NA
$C_{10}H_{12}O_3F_{10}S$	402.03476	401→320	6.80 ± 0.13	1,1,2,2,3,3,4,4,5,5-Decafluoro-decane-1-sulfonic acid (5:5 H-FTDSA)	5.00	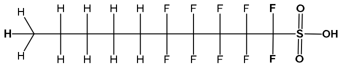	RN:1980050-03-0	62367887

$C_{12}H_{14}O_3F_{12}S$	466.0472	465→384	4.18 ± 0.06	1,1,2,2,3,3,4,4,5,5,6,6-Dodecafluoro-dodecane-1-sulfonic acid (6:6 H-FTDoSA)	6.16	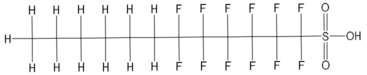	NA	NA
$C_{16}H_{18}O_3F_{16}S$	594.07211	593→512	1.29 ± 0.05	1,1,2,2,3,3,4,4,5,5,6,6,7,7,8,8-Hexadecafluoro-hexadecane-1-sulfonic acid (8:8 H-FTHxDSA)	8.48	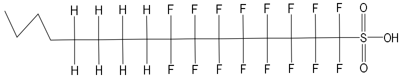	NA	NA
**III. Ether-PFAA**								
**Class5 H-PFOiCA**								
$C_4H_2O_3F_6$	211.99081	211→167	22.07 ± 2.15	Difluoro(1,1,2,2-tetrafluoroethoxy)acetic acid (H-PFOPeA)	1.67	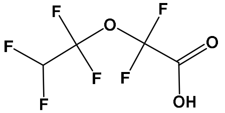	RN:81233-13-8	35788312
$C_5H_2O_3F_8$	261.98761	261→217	18.95 ± 2.01	2,2,3,4,4-Pentafluoro-4-(trifluoromethoxy)butanoic acid (H-PFOHxA)	2.64	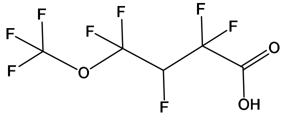	RN:919005-24-6	28571971
$C_6H_2O_3F_{10}$*	311.98444	311→267	7.24 ± 1.18	2,2,3,4,4-Pentafluoro-4-(pentafluoroethoxy)butanoic acid (H-PFOHpA)	3.6	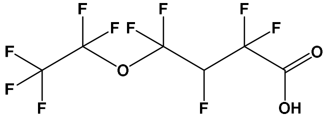	RN:919005-25-7	28571969
$C_8H_2O_3F_{14}$*	411.97803	411→367	145.49 ± 9.27	(1,1,2,2,3,3,4,4,5,5,6,6-Dodecafluoro-hexyloxy)-difluoro-acetic acid (H-PFONA)	4.35	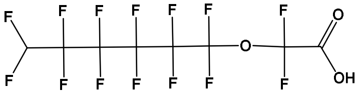	NA	NA
$C_9H_2O_5F_{16}$	493.96467	493→449	7.83 ± 0.97	{[(1,1,2,2,3,3,4,4,5,5-Decafluoro-pentyloxy)-difluoro-methoxy]-difluoro-methoxy}-difluoro-acetic acid (H-PFO3DOA)	6.38	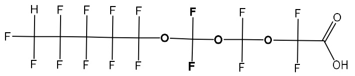	NA	NA
$C_9H_2O_6F_{16}$	509.95959	509→465	1.02 ± 0.04	({[Difluoro-(1,1,2,2,3,3,4,4-octafluoro-butoxy)-methoxy]-difluoro-methoxy}-difluoro-methoxy)-difluoro-acetic acid (H-PFO4TrDA)	6.95	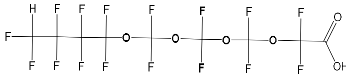	NA	NA
$C_{10}H_2O_4F_{18}$	527.96656	527→483	26.75 ± 3.63	[Difluoro-(1,1,2,2,3,3,4,4,5,5,6,6,7,7-tetradecafluoro-heptyloxy)-methoxy]-difluoro-acetic acid (H-PFO2DOA)	6.33	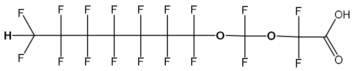	NA	NA
$C_{11}H_2O_4F_{20}$*	577.96338	577→533	51.27 ± 2.82	[Difluoro-(1,1,2,2,3,3,4,4,5,5,6,6,7,7,8,8-hexadecafluoro-octyloxy)-	7	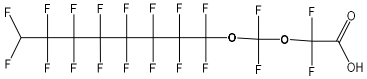	NA	NA

$C_{11}H_2O_6F_{20}$	609.95320	609→565	7.81 ± 0.55	methoxy]-difluoro-acetic acid (H-PFO2TrDA) ({[(1,1,2,2,3,3,4,4,5,5,6,6-Dodecafluoro-hexyloxy)-difluoro-methoxy]-difluoro-methoxy}-difluoro-methoxy)-difluoro-acetic acid (H-PFO4PeDA)	8.29	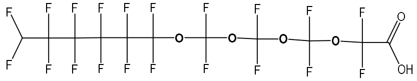	NA	NA
				**Classs6 PFOiCA**				
$C_3HO_3F_5$*	179.98459	179→135	44.32 ± 2.69	Difluoro(trifluoromethoxy)acetic acid (PFOBA)	1.31	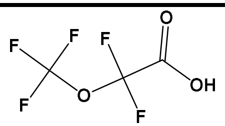	RN:674-13-5	4299802
$C_4HO_3F_7$	229.98138	229→185	5.95 ± 0.50	Perfluoromethoxypropionic acid (PFOPeA)	2.28	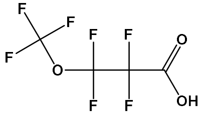	RN:377-73-1	107331
$C_4HO_4F_7$	245.97630	245→201	3.34 ± 0.48	[Difluoro(trifluoromethoxy)methoxy] (difluoro)acetic acid (PFO2HxA)	2.63	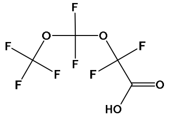	NA	58539806
$C_5HO_3F_9$*	279.97821	279→235	15.36 ± 1.77	2,2,3,3-Tetrafluoro-3-(pentafluoroethoxy)propanoic acid (PFOHxA)	3.24	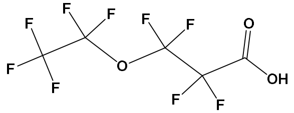	RN:377-76-4	10570931
$C_5HO_4F_9$	295.97311	295→251	0.61 ± 0.06	Difluoro[1,1,2,2-tetrafluoro-2-(trifluoromethoxy)ethoxy]acetic acid (PFO2HpA)	3.09	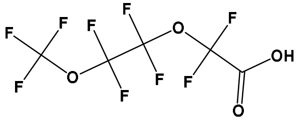	RN:151772-58-6	2062549
$C_5HO_5F_9$	311.96801	311→267	0.95 ± 0.08	{[Difluoro(trifluoromethoxy)methoxy](difluoro)methoxy}(difluoro)acetic acid (PFO3OA)	9.94	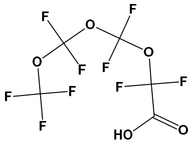	RN:39492-89-2	35801757
$C_6HO_4F_{11}$*	345.96991	345→301	53.26 ± 3.53	PERFLUORO-3,6-DIOXAOCTANOIC ACID (PFO2OA)	4.05	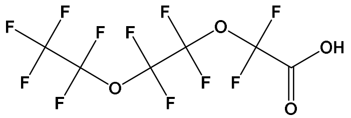	RN:80153-82-8	9741939
$C_7HO_3F_{13}$*#	379.97180	379→335	16.83 ± 1.85	2,2,3,3,4,4-Hexafluoro-4-(heptafluoropropoxy)butanoic acid (PFOOA)	4.58	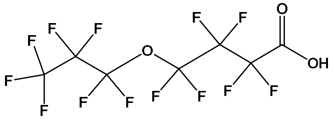	RN:801212-59-9	95788262

$C_8HO_3F_{15}$*	429.96863	429→385	496.96 ± 4.79	2,2,3,3,4,4-Hexafluoro-4-(nonafluorobutoxy)butanoic acid (PFONA)	5.25	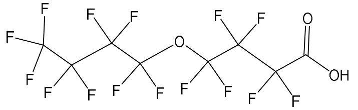	NA	95746076
$C_8HO_5F_{15}$	461.95844	461→417	6.95 ± 1.45	[(Difluoro-nonafluorobutyloxy-methoxy)-difluoro-methoxy]-difluoro-acetic acid (PFO3UNDA)	5.68	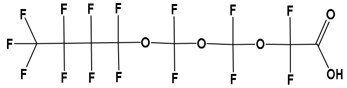	NA	NA
$C_8HO_8F_{15}$	509.94319	509→465	2.23 ± 0.90	[({[(Difluoro-trifluoromethoxy-methoxy)-difluoro-methoxy]-difluoro-methoxy}-difluoro-methoxy)-difluoro-methoxy]-difluoro-acetic acid (PFO6TEDA)	7.88	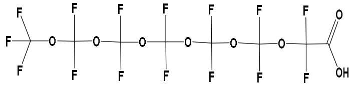	NA	NA
$C_9HO_3F_{17}$*[#]	479.96542	479→435	43.83 ± 2.68	Difluoro-pentadecafluoroheptyloxy-acetic acid (PFODA)	5.62	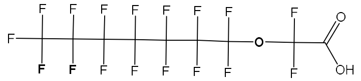	NA	NA
$C_9HO_5F_{17}$*	511.95525	511→467	20.40 ± 2.00	[(Difluoro-undecafluoropentyloxy-methoxy)-difluoro-methoxy]-difluoro-acetic acid (PFO3DOA)	6.91	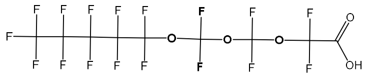	NA	NA
**Class7 HFPO**								
$C_6HO_3F_{11}$*[#]	329.97501	329→285	51.08 ± 3.71	2,2,3,3-Tetrafluoro-3-(heptafluoropropoxy)propanoic acid (HFPO-DA, GenX)	3.91	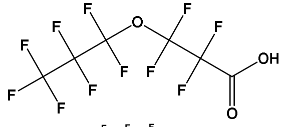	CS:206-822-8 RN:378-03-0	2282437
$C_7HO_4F_{13}$	395.96674	395→351	2.50 ± 0.18	2,2,3,3-Tetrafluoro-3-[1,1,2,2,3,3-hexafluoro-3-(trifluoromethoxy)propoxy]propanoic acid (HFPO-TA(C7))	4.72	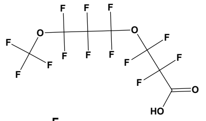	NA	58539807
$C_8HO_4F_{15}$*	445.96353	445→401	52.76 ± 2.84	Difluoro[1,1,2,2-tetrafluoro-2-(nonafluorobutoxy)ethoxy]acetic acid (HFPO-TA(C8))	5.39	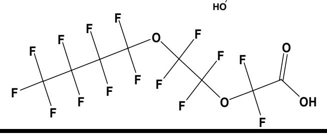	RN:13778069-9	2058926
**Class8 n:2 FTOiCA**								
$C_8H_3O_3F_{13}$	393.98746	393→349	8.42 ± 1.60	Difluoro-(1,1,2,2,3,3,4,4,5,5,6-undecafluoro-hexyloxy)-acetic acid (6:2 FTONA)	4.78	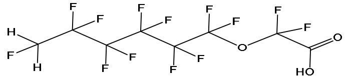	RN:759414-86-3	28573402
$C_9H_3O_4F_{15}$	459.97919	459→415	0.64 ± 0.03	[Difluoro-(1,1,2,2,3,3,4,4,5,5,6-undecafluoro-hexyloxy)-methoxy]-difluoro-acetic acid (7:2 FTO2UNDA)	6.09	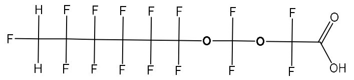	RN:129301-40-2	2049881

$C_{12}H_3O_7F_{21}$*	657.95434	657→613	148.82 ± 3.74	[({[Difluoro-(1,1,2,2,3,3,4,4,5,5,6-undecafluoro-hexyloxy)-methoxy]-difluoro-methoxy}-difluoro-methoxy)-difluoro-methoxy]-difluoro-acetic acid (10:2 FTO5HpDA)	10.03	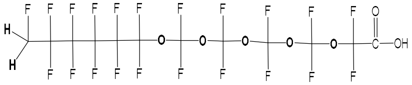	NA	NA
**Class9 n:m H-FTOiSA**								
$C_4H_4O_4F_6S$	261.97345	261→181	0.93 ± 0.66	Difluoro-(2,3,3,3-tetrafluoro-propoxy)-methanesulfonic acid (3:1 H-FTPOPeSA)	8.29	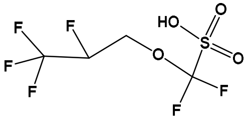	NA	NA
$C_5H_4O_4F_8S$	311.97025	311→231	4.92 ± 0.34	2,2,3,3,4,4,5,5-Octafluoropentyl hydrogen sulfate (4:1 H-FTOHxSA)	0.43	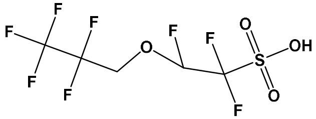	NA	NA
$C_6H_6O_4F_8S$	325.98590	325→245	2.71 ± 0.55	1,1,4,5,5-Pentafluoro-5-trifluoromethoxy-pentane-1-sulfonic acid (4:2 H-FTOHpSA)	2.63	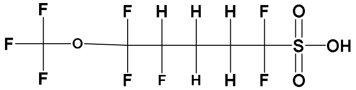	NA	NA
$C_6H_8O_4F_6S$*	290.00476	289→208	22.62 ± 2.11	1,1,5-Trifluoro-5-trifluoromethoxy-pentane-1-sulfonic acid (3:3 H-FTOHpSA)	2.76	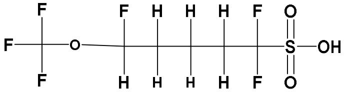	RN:1357625-09-2	NA
$C_8H_8O_4F_{10}S$	389.99836	389→309	5.31 ± 1.30	1,1,2,2,3,3,4,4,5,5-Decafluoro-5-propoxy-pentane-1-sulfonic acid (5:3 H-FTONSA)	3.31	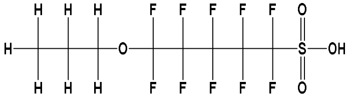	NA	NA
$C_8H_{10}O_4F_8S$	354.0172	353→272	47.71 ± 1.14	1,1,2,2,3,3,4,4-Octafluoro-5-propoxy-pentane-1-sulfonic acid (4:4 H-FTONSA)	1.31	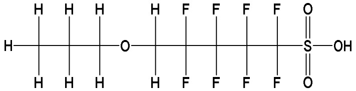	NA	NA
$C_8H_{10}O_5F_8S$	370.01212	369→288	2.51 ± 0.17	1,1,2,2,3,3,4,4-Octafluoro-4-propoxymethoxy-butane-1-sulfonic acid (4:4 H-FTO2DSA)	2.38	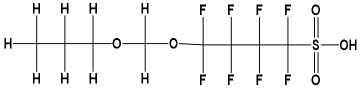	NA	NA
$C_9H_8O_4F_{12}S$	439.99517	439→359	2.51 ± 0.18	1,1,2,2,3,3,4,4,5,5,6,6-Dodecafluoro-6-propoxy-hexane-1-sulfonic acid (6:3 H-FTODSA)	3.98	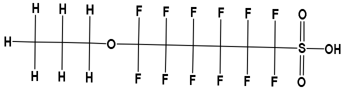	NA	NA
$C_{10}H_{12}O_4F_{10}S$	418.02966	417→336	32.69 ± 0.26	Difluoro-(1,1,2,2,3,3,4,4-octafluoro-nonyloxy)-methanesulfonic acid (5:5 H-FTOUNDSA)	5.14	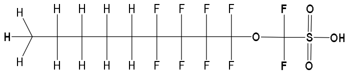	NA	NA

$C_{12}H_{12}O_4F_{14}S$	518.02327	517→436	2.92 ± 0.13	(1,1,2,2,3,3,4,4,5,5,6,6-Dodecafluoro-undecyloxy)-difluoro-methanesulfonic acid (7:5 H-FTOTrDSA)	6.48	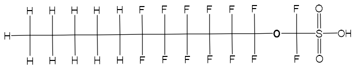	NA	NA
$C_{13}H_{12}O_4$F16 S	568.02008	567→486	1.60 ± 0.09	Difluoro-(1,1,2,2,3,3,4,4,5,5,6,6,7,7-tetradecafluoro-dodecyloxy)-methanesulfonic acid (8:5 H-FTOTEDSA)	7.15	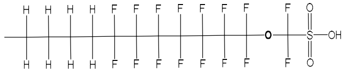	NA	NA
$C_{14}H_{16}O_4F_{14}S$	546.05457	545→464	9.46 ± 0.24	(1,1,2,2,3,3,4,4,5,5,6,6-Dodecafluoro-tridecyloxy)-difluoro-methanesulfonic acid (6:7 H-FTOPeDA)	7.46	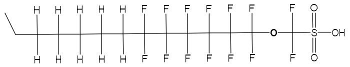	NA	NA
$C_{16}H_{18}O_4F_{16}S$	610.06703	609→528	2.98 ± 0.56	Difluoro-(1,1,2,2,3,3,4,4,5,5,6,6,7,7-tetradecafluoro-pentadecyloxy)-methanesulfonic acid (8:8 H-FTOHpDA)	8.62	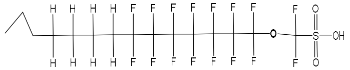	NA	NA
**Class10 kU-PFOiCA**								
$C_5HO_3F_7$*#	241.98138	241→197	42.32± 4.13	2,2,3,3-Tetrafluoro-3-[(trifluorovinyl)oxy]propanoic acid (U-PFOHxA)	1.7	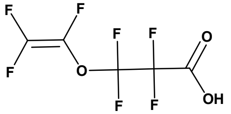	NA	9405881
$C_6HO_3F_9$*	291.97821	291→247	17.96 ± 2.02	Difluoro{[(2E)-1,1,2,3,4,4,4-heptafluoro-2-buten-1-yl]oxy}acetic acid (U-PFOHpA)	3.15	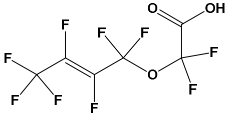	NA	95752668
$C_7HO_3F_{11}$	341.97500	341→297	19.38 ± 1.43	2,2,3,3,4,4-Hexafluoro-4-pentafluoroallyloxy-butyric acid (U-PFOOA)	3.88	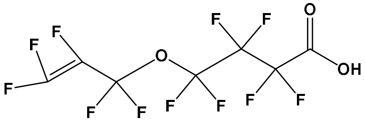	NA	NA
$C_8HO_3F_{11}$	353.97500	353→309	1.18 ± 0.24	2,2,3,3-Tetrafluoro-3-heptafluoropenta-2,4-dienyloxy-propionic acid (2U-PFONA)	4.09	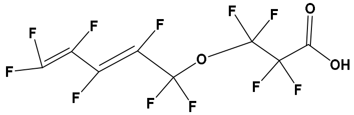	NA	NA
$C_8HO_3F_{13}$*#	391.97180	391→347	1207.83 ± 66.88	2,2,3,3-Tetrafluoro-3-nonafluoropent-4-enyloxy-propionic acid (U-PFONA)	4.85	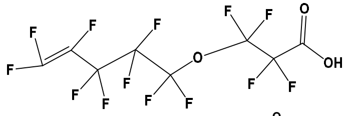	NA	9935695
$C_8HO_4F_{13}$	407.96674	407→363	11.36 ± 2.09	2,2,3,3-Tetrafluoro-3-{1,1,2,3,3,3-hexafluoro-2-[(trifluorovinyl)oxy]propoxy}propanoic acid (U-PFO2DA)	3.89	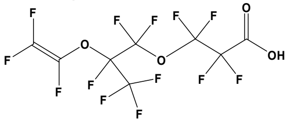	NA	95721746

$C_8HO_5F_{13}$	423.96164	423→379	2.89 ± 0.31	[(Difluoro-heptafluorobut-3-enyloxy-methoxy)-difluoro-methoxy]-difluoro-acetic acid (U-PFO3UNDA)	5.84	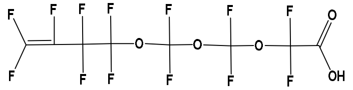	NA	NA
$C_9HO_3F_{15}$*	441.96861	441→397	7.07 ± 1.10	Difluoro-tridecafluorohept-6-enyloxy-acetic acid (U-PFODA)	5.21	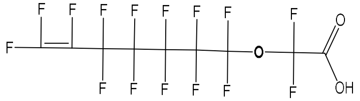	NA	NA
$C_7HO_4F_{11}$	357.96992	357→313	4.97 ± 4.97	Difluoro-(1,2,2,2-tetrafluoro-1-pentafluoroallyloxy-ethoxy)-acetic acid (U-PFO2NA)	3.42	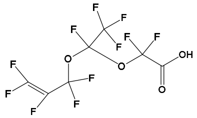	NA	NA
**Class11 PFOiAA**								
$C_5H_2O_6F_8S$	341.94443	341→261	3.59 ± 0.40	2,3,3,3-Tetrafluoro-2-(1,1,2,2-tetrafluoro-2-sulfoethoxy)propanoic acid (PFOHxAA)	1.02	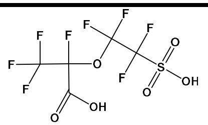	NA	9200649
$C_8H_2O_7F_{14}S$	507.92978	507→427	0.59 ± 0.07	2,3,3,3-Tetrafluoro-2-[1,1,2,3,3,3-hexafluoro-2-(1,1,2,2-tetrafluoro-2-sulfoethoxy)propoxy]propanoic acid (PFO2DA)	3.21	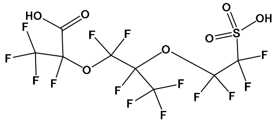	NA	95689763
**IV. Cl-PFCA**								
**Class12 Cl-PFCA**								
$C_2HO_2ClF_2$	129.96332	129→85	2.26 ± 0.18	Chlorodifluoroacetic acid (Cl-TFA)	0.82	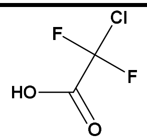	CS:200-928-8 RN:76-04-0	10800462
$C_7HO_2ClF_{12}$	379.94736	379→335	8.48 ± 0.64	7-Chlorododecafluoroheptanoic acid (Cl-PFHpA)	4.46	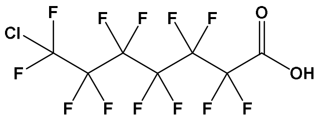	RN:1550-24-9	2018292
$C_3HO_2ClF_4$*	179.96011	179→135	99.67 ± 3.38	3-Chlorotetrafluoropropionic acid (Cl-PFPrA)	1.78	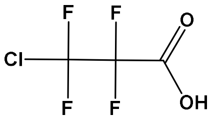	RN:661-82-5	2062646
$C_4HO_2ClF_6$*[#]	229.95692	229→185	113.68 ± 3.51	4-Chloro-2,2,3,3,4,4-hexafluorobutanoic acid (Cl-PFBA)	2.45	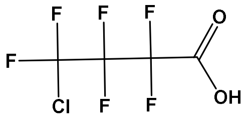	RN:661-82-5	2345668

$C_5HO_2ClF_8$*	279.95374	279→235	65.64 ± 3.03	5-Chloro-2,2,3,3,4,4,5,5-octafluoropentanoic acid (Cl-PFPeA)	3.12	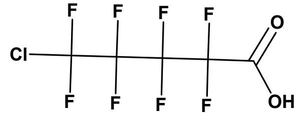	RN:3110-03-0	2050153
$C_6HO_2ClF_{10}$*#	329.95053	329→285	49.23 ± 3.79	6-Chloro-2,2,3,3,4,4,5,5,6,6-decafluorohexanoic acid (Cl-PFHxA)	3.79	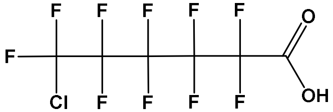	NA	2862278
$C_8HO_2ClF_{14}$*#	429.94415	429→385	49.11 ± 4.39	8-Chloro-2,2,3,3,4,4,5,5,6,6,7,7,8,8-tetradecafluorooctanoic acid (Cl-PFOA)	5.13	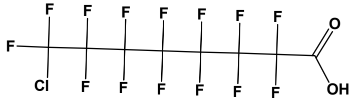	RN:335-63-7	2283188
**Class13 Cl-PFOiCA**								
$C_7HO_4ClF_{12}$	411.93716	411→367	4.11 ± 0.32	[1-(3-Chloro-1,1,2,2,3,3-hexafluoropropoxy)-1,2,2,2-tetrafluoroethoxy](difluoro)acetic acid (Cl-PFO2NA)	4.44	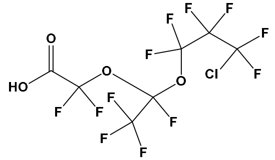	NA	95776619
$C_9HO_5ClF_{16}$	527.9257	527→483	20.77 ± 2.21	{1-[1-(3-Chloro-1,1,2,2,3,3-hexafluoropropoxy)-1,2,2,2-tetrafluoroethoxy]-1,2,2,2-tetrafluoroethoxy}(difluoro)acetic acid (Cl-PFO3DOA)	5.62	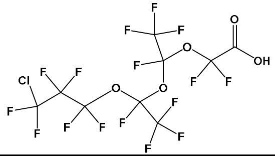	NA	95653019
**V. I-PFAA**								
**Class14 n:(m+1) I-FTCA**								
$C_6H_5O_2F_6I$*#	349.92385	349→304	33.20 ± 2.76	4,4,5,5,6,6-Hexafluoro-6-iodo-hexanoic acid (3:3 I-FTHxA)	3.94	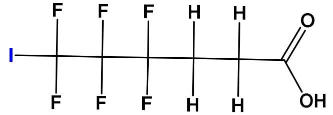	NA	NA
$C_8H_7O_2F_8I$*#	413.9363	413→368	34.98 ± 3.01	3,3,5,5,6,6,7,7-Octafluoro-8-iodo-octanoic acid (4:4 I-FTOA)	5.39	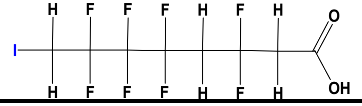	NA	NA
**Class15 n:m I-FTOSA**								
$C_2H_3O_4F_2IS$*	287.87648	287→206	170.16 ± 12.01	Difluoro-iodomethoxy-methanesulfonic acid (1:1 I-FTOPrSA)	0.12	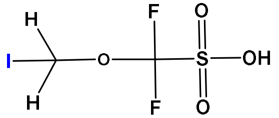	NA	NA

$C_4H_5O_4F_4IS$*#	351.88894	351→270	42.18 ± 3.02	Difluoro-iodomethoxy-methanesulfonic acid (2:2 I-FTOPeSA)	1.22	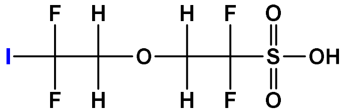	NA	NA
$C_6H_7O_4F_6IS$*	415.9014	415→334	48.98 ± 1.53	Difluoro-(1,1,5,5-tetrafluoro-5-iodo-pentyloxy)-methanesulfonic acid (3:3 I-FTOHpSA)	2.48	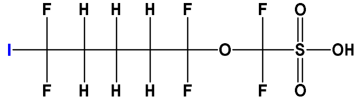	NA	NA

Note: *51 PFAS with concentrations higher than 10 μg L^{-1}; #23 PFAS in breakthrough curves study.

Supplementary Table 2. The water quality characteristics of the collected fluorochemical effluent samples**.**

Water parameters	
pH	7.3 ± 0.1
∑PFAS (mg L^{-1})	36 ± 1.99
TOC (mg L^{-1})	35.86 ± 0.43
TIC (mg L^{-1})	26.22 ± 0.44
TOF (mg L^{-1})	49.05 ± 2.91
Cl^- (mg L^{-1})	388.42 ± 2.34
F^- (mg L^{-1})	44.52 ± 1.22
NO_3^- (mg L^{-1})	34.51 ± 0.92
SO_4^{2-} (mg L^{-1})	181.07 ± 4.18

Concentrations total PFAS (∑PFAS) were measured by liquid chromatography Orbitrap mass spectrometry. Concentrations of total organic carbon (TOC) and total inorganic carbon (TIC) were measured by a multi N/C UV TOC analyzer. Concentrations of total organic fluorine (TOF) and anion were measured by combustion-ion chromatography. All the error bars in this table represent the standard deviation of the data from duplicate tests.

Supplementary Table 3. Maximum adsorption amount in PFAS simulated water by Zn-based EC.

PFAS type	log K_{ow}	q_m (mmol g^{-1} Zn hydroxide flocs)
PFBA	2.14	< 0.1
GenX	3.36	1.6 ± 0.7
PFHxA	3.48	0.6 ± 0.3
PFHpA	4.15	4.6 ± 0.6
PFOA	4.81	6.4 ± 0.4
PFOS	4.88	7.5 ± 0.04
PFNA	5.48	11.1 ± 1.7
PFDA	6.15	> 23

The log K_{ow} values were estimated by the EPI Suite software. The q_m values (mmol g^{-1} Zn hydroxide flocs) were detected under following conditions: initial PFAS concentration of 1.2mM, current density of 1.0 mA cm^{-2}, electrolyte concentration of 20 mM NaCl, pH=7. All the error bars in this table represent the standard deviation of the data from duplicate tests.

Supplementary Table 4. Comparison of PFOA adsorption performance in the reported literature (n=23).

Adsorbent	C_0 (mM)	q_m (mmol g^{-1})	t_{eq} (h)	q_{dyn} (mmol g^{-1} h^{-1})	References
GAC	0.12	0.39	144	2.7×10^{-3}	Yu et al.[1]
PAC		0.67	2	0.34	
AI400 (IXR)		2.92	120	2.4×10^{-2}	
PAC	1.69	1.17	3	0.39	Li et al.[2]
GAC	0.29	1	36	2.7×10^{-2}	Du et al.[3]
IRA67 (IXR)		2	24	8.3×10^{-2}	
A600E (IXR)	2.42	0.3	60	5×10^{-3}	Zaggia et al.[4]
A520E (IXR)		0.33	60	5.4×10^{-3}	
A532E (IXR)		0.34	60	5.7×10^{-3}	
AC	0.06	0.018	1	1.8×10^{-2}	This work
PFA694E (IXR)		0.15	1	0.15	
Fe-BTC	1.21	0.98	100	1.2×10^{-2}	Yang et al.[5]
MIL-96-RHPAM2 (MOF)	2.42	0.82	186	4.4×10^{-3}	Mohd et al.[6]
UiO-66 (MOF)	1.21	0.8	1	0.8	Sini et al.[7]
MIL-101(Cr) (MOF)	0.24	1.16	1	1.16	Liu et al[8]
MIL-53 (Al) (MOF)	0.002	0.41	4	0.1	Jun et al.[9]
UiO-67 (MOF)	1.21	1.14	1	1.14	Clark et al.[10]
Carbon nanotubes (CNT)	0.2	0.006	8	8.1×10^{-4}	Li et al.[11]
CNT	0.12	0.32	10	3.2×10^{-2}	Li et al[12]
β-cyclodextrin	0.03	0.082	13.5	6×10^{-3}	Xiao et al.[13]
NH_2-β-cyclodextrin	0.48	1.10	48	2.3×10^{-2}	Yang et al.
Zn-based EC	1.2	6.4	0.03	213.3	This work
Zn-based EC	0.8	6.05	0.03	201.7	Lin et al.[14]

In this table, C_0 refers to the initial concentration (mM); q_m refers to the maximum adsorption amount (mmol g^{-1}); t_{eq} refers to the equilibrium time (h); q_{dyn} refers to the dynamic adsorption capacity (mmol g^{-1} h^{-1}), $q_{dyn} = q_m/t_{eq}$; GAC refers to the granular activated carbon; PAC refers to the powdered activated carbon. IXR refers to the ion exchange resin; MOF refers to the metal-organic framework; CNT refers to the carbon nanotubes; Zn-based EC refers to the zinc-based electrocoagulation.

Supplementary Table 5. Comparison of PFOS adsorption performance in the reported literature (n=25).

Adsorbent	C_0 (mM)	q_m (mmol g^{-1})	t_{eq} (h)	q_{dyn} (mmol g^{-1} h^{-1})	References
GAC		0.37	144	2.6×10^{-3}	
PAC	0.12	1.04	2	0.52	Yu et al.[1]
AI400(IXR)		0.42	120	3.5×10^{-3}	
GAC	0.02	0.14	24	5.83×10^{-3}	Zhang et al.[15]
F300 (GAC)	0.3	0.39	48	8.12×10^{-3}	
URV-MOD-1 (GAC)	0.3	0.47	48	9.79×10^{-3}	Ochoa-Herrera et al.[16]
F400 (GAC)	0.3	0.423	48	8.81×10^{-3}	
GAC	2	0.12	8	1.5×10^{-2}	Zhang et al.[17]
A520E (IXR)	0.2	0.435	24	1.81×10^{-2}	Shahrokhi et al.[18]
PAC	0.2	0.37	0.08	4.63	
A300 (IXR)		0.27	48	5.63×10^{-3}	
A520E (IXR)		1.36	48	2.83×10^{-2}	
A600E (IXR)	9.78×10^{-2}	0.26	48	5.42×10^{-3}	Fang et al.[19]
A860 (IXR)		0.88	48	1.83×10^{-2}	
CalRes2301 (IXR)		0.66	48	1.38×10^{-2}	
NU-1000 (MOF)	0.2	1.24	0.016	77.5	Li et al.[20]
MIL-101(Cr) (MOF)	1	0.75	1	0.75	Liu et al.[8]
UiO-66 (MOF)	1	0.35	1	0.35	Clark et al.[10]
CNT	1	1.1	2	0.55	Niu et al.[21]
	0.2	1.4	2	0.7	
CNT	0.02	0.01	2	5.0×10^{-3}	Li et al.[22]
β-cyclodextrin	0.01	0.145	6	2.4×10^{-2}	Ching et al.[23]
β-cyclodextrin	3.3×10^{-6}	3.18×10^{-3}	9	3.53×10^{-4}	Wang et al.[24]
Zn-based EC	0.6	7.47	0.07	106.7	This work
Zn-based EC	0.5	7.17	0.05	143.4	Lin et al.[14]

In this table, C_0 refers to the initial concentration (mM); q_m refers to the maximum adsorption amount (mmol g^{-1}); t_{eq} refers to the equilibrium time (h); q_{dyn} refers to the dynamic adsorption capacity (mmol g^{-1} h^{-1}), $q_{dyn} = q_m/\underline{t}_{eq}$; GAC refers to the granular activated carbon; PAC refers to the powdered activated carbon. IXR refers to the ion exchange resin; MOF refers to the metal-organic framework; CNT refers to the carbon nanotubes; Zn-based EC refers to the zinc-based electrocoagulation.

Supplementary Table 6. Comparison of GenX adsorption performance in the reported literature (n=10).

Adsorbent	C_0 (mM)	q_m (mmol g^{-1})	t_{eq} (h)	q_{dyn} (mmol g^{-1} h^{-1})	References
NH_2-β-cyclodextrin	0.66	0.73	2	0.37	Yang et al.[25]
β-cyclodextrin	3.0×10^{-3}	0.015	2	7.5×10^{-3}	
GAC	0.152	0.79	20	0.040	Wang et al.[26]
PAC		0.79	20	0.040	
IRA400 (IXR)		2.78	72	0.039	
IRA67 (IXR)		3.22	48	0.067	
MOF	6.7×10^{-3}	0.16	1.5	0.11	Guo et al.[27]
GAC	6.1×10^{-4}	3.27×10^{-3}	12	2.72×10^{-4}	Tan et al.[28]
PAC		4.57×10^{-3}	1	4.57×10^{-3}	
Zn-based EC	1.2	1.56	0.07	22.29	This work

In this table, C_0 refers to the initial concentration (mM); q_m refers to the maximum adsorption amount (mmol g^{-1}); t_{eq} refers to the equilibrium time (h); q_{dyn} refers to the dynamic adsorption capacity (mmol g^{-1} h^{-1}), $q_{dyn} = q_m/t_{eq}$; GAC refers to the granular activated carbon; PAC refers to the powdered activated carbon. IXR refers to the ion exchange resin; MOF refers to the metal-organic framework; Zn-based EC refers to the zinc-based electrocoagulation.

Supplementary Table 7. F/Zn atomic ratios of different PFAS-adsorbed zinc hydroxide flocs.

F(atom)/Zn(atom)	XPS	EDX
Zn hydrophobic flocs	0	0
PFBA- Zn hydrophobic flocs	0.91	0.08
PFHxA- Zn hydrophobic flocs	1.04	0.29
PFHpA- Zn hydrophobic flocs	4.27	4.66
PFOA- Zn hydrophobic flocs	6.35	7.54
PFNA- Zn hydrophobic flocs	7.90	7.35
PFDA- Zn hydrophobic flocs	8.85	9.96

The zinc hydroxide flocs in-situ by Zn-based EC in PFAS simulated water under following conditions: initial PFAS concentration of 1.2mM, 1 mA cm^{-2} of applied current density, 20 mM NaCl of electrolyte concentration, pH=7. The zinc hydroxide flocs were freeze-dried before characterizations. Percentages of F and Zn atomic on the surface of zinc hydroxide flocs was detected by X-ray Photoelectron Spectroscopy (XPS) and Energy Dispersive Spectrometer (EDX).

Supplementary Table 8. Properties of PFA694E.

Parameters	PFA694E (Crushed)	PFA694E (As-received)
Mean diameter (μm)	675	214 ± 40
Total anion capacity (mmol Cl^{-1} per mL resin)	5.85 ± 0.35	6.58 ± 0.36

The total anion exchange capacities of the crushed and as-received PFA694E resins were measured using the GB/T 11992-2008 National Standard of China in polypropylene columns. All the error bars in this table represent the standard deviation of the data from duplicate tests.

Supplementary Table 9. Design parameters for RSSCT.

Parameters	AC	PFA694E
$EBCT_{LC}$ (min)	7.57	4.78
$EBCT_{SC}$ (min)	0.83	0.83
d_{LC} (mm)	0.85	0.675
d_{SC} (mm)	0.282	0.282
Flow rate $_{SC}$ (mL min^{-1})	2	2
Sorbent volume (cm^3)	1.03	0.93

In this table, ECBT refers to the empty bed contact time (min); SC and LC refer to the RSSCT column and full-scale adsorber, respectively; *d* represents the diameter of the adsorbent (mm).

Supplementary Table 10. Carbon emissions and treatment costs.

		PFA694E bed alone		Treatment-train	
		99% PFOA	90% PFOA	99% PFOA	90% PFOA
Solid generation (kg)	PFA694E	2.60	1.73	0.30	0.11
	Zinc hydroxide flocs	/	/	0.55	0.55
	ZnO	/	/	0.45	0.45
CO_2 equipment (kg)	PFA694E	9.71	6.45	1.12	0.40
	Zn metal	/	/	0.98	0.98
	Electricity	/	/	0.58	0.58
	Spent adsorbents incineration (PFA694E/ Zinc hydroxide flocs)	10.99	7.30	1.26/1.34	0.46/1.34
	Total	20.70	13.75	5.28	3.76
Cost (dollar)	PFA694E	74.38	49.39	8.54	3.08
	Zn metal	/	/	1.14	1.14
	Electricity	/	/	0.10	0.10
	Spent adsorbents incineration (PFA694E/ Zinc hydroxide flocs)	0.82	0.54	0.09/0.17	0.03/0.17
	Total	75.10	49.93	10.04	4.52

Supplementary Table 11. Information of MPFAC-C-ES internal standards.

Internal standards	Concentration in MeOH （ng mL^{-1}）	m/z
MPFBA	2000	217
M5PFPeA	2000	268
M5PFHxA	2000	318
M4PFHpA	2000	367
M8PFOA	2000	421
M9PFNA	2000	472
M6PFDA	2000	519
M7PFUdA	2000	570
MPFDoA	2000	615
M2PFTeDA	2000	715
M3PFBS	2000 (As the salt)	302
M3PFhXS	2000 (As the salt)	402
M8PFOS	2000 (As the salt)	507

Supplementary Fig. 1. Photos of the experimental systems. a, b Photos of the electrocoagulation (**a**) and RSSCT (**b**) setup.

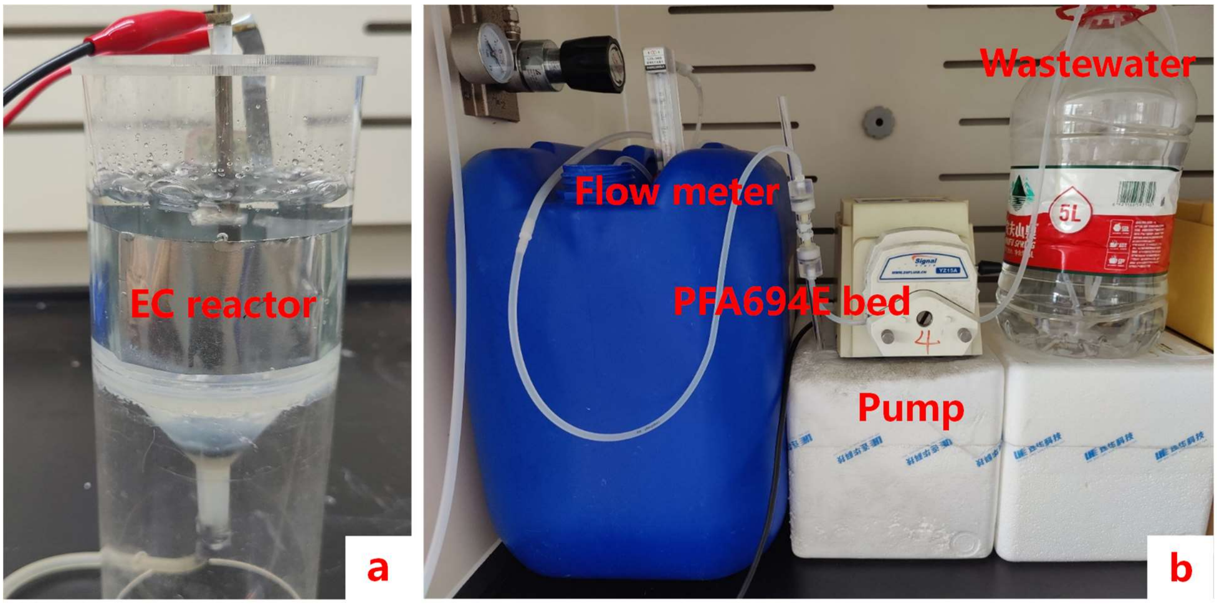

Supplementary Fig. 2. Sorption isotherms of PFOA and GenX on the PFA694E and AC. Adsorption isotherm tests were conducted by AC and PFA694E with PFOA solution of a wide range from 10 to 400 mg L^{-1} (AC) or 100 to 600 mg L^{-1} (PFA694E) for 24 h under 25°C. pH=7. All isotherm data were fitted by the Langmuir model.

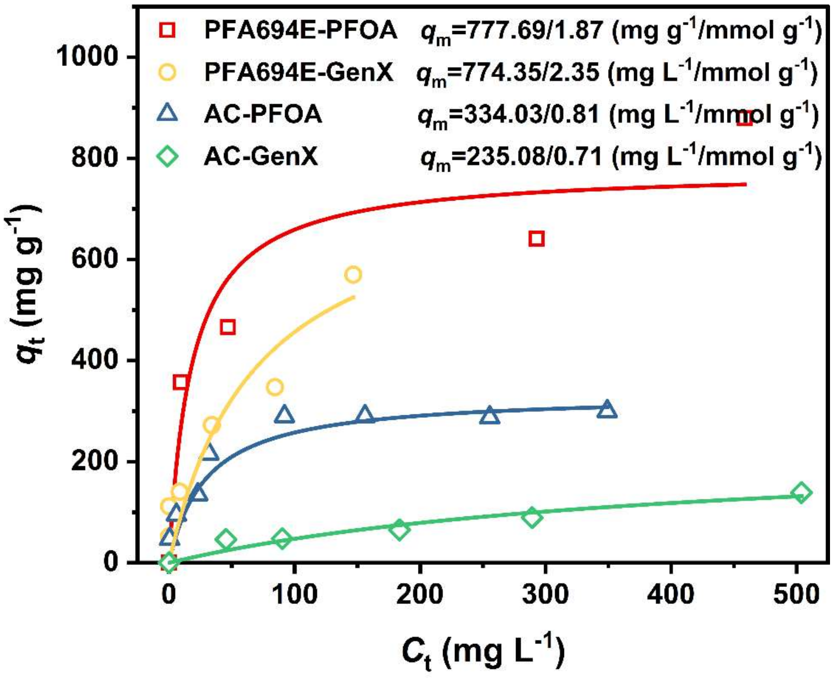

Supplementary Fig. 3. Performance of different systems for the removal of 9 PFCA from fluorochemical wastewater. Experiment conditions: 100 cm^2 of Zn electrode, 300 mL fluorochemical wastewater, 3 mA cm^{-2} of applied current density.

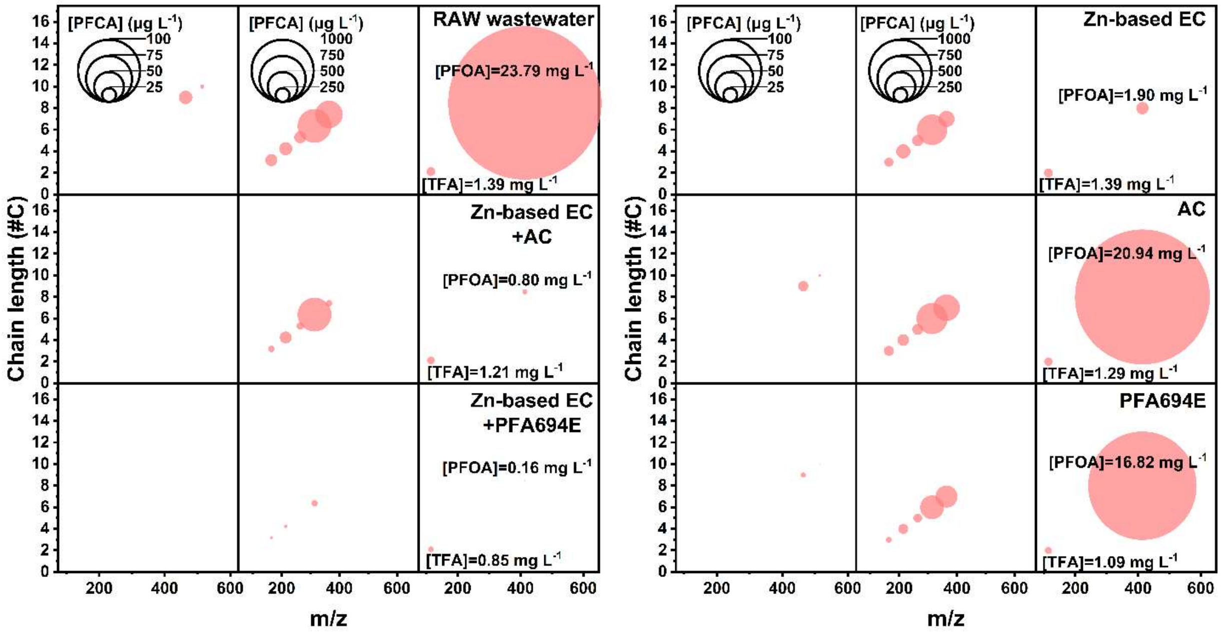

Supplementary Fig. 4. Performance of different systems for the removal of 14 Cl/I-PFAA from fluorochemical wastewater. Experiment conditions: 100 cm^2 of Zn electrode, 300 mL fluorochemical wastewater, 3 mA cm^{-2} of applied current density.

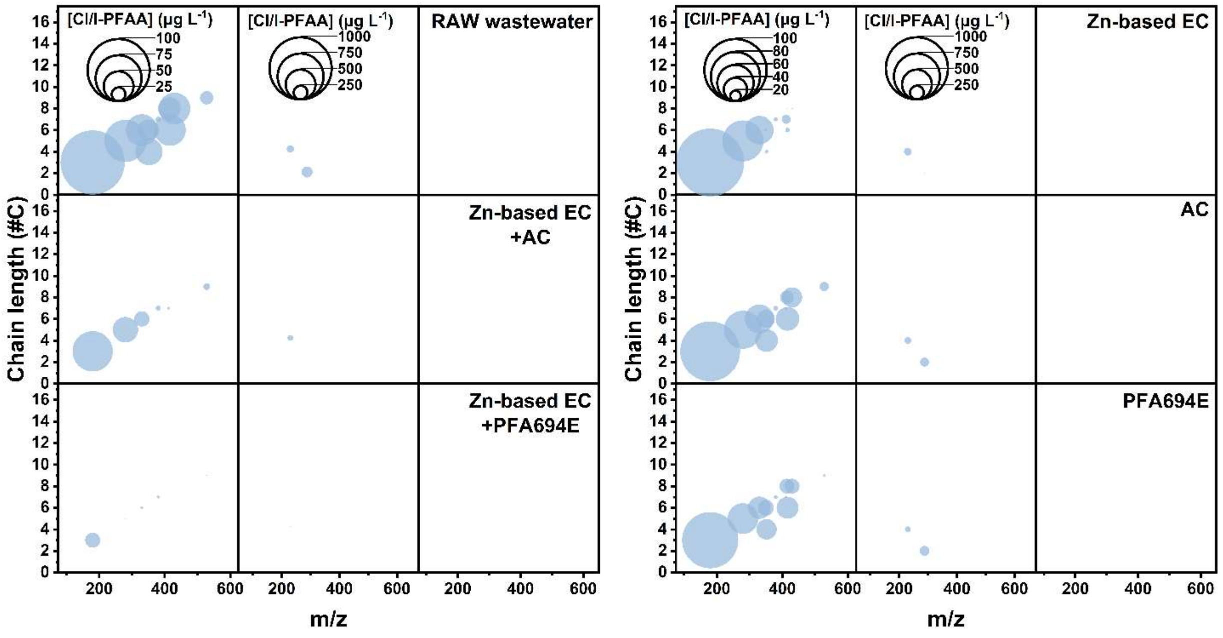

Supplementary Fig. 5. Performance of different systems for the removal of 52 Ether-PFAA from fluorochemical wastewater. Experiment conditions: 100 cm^2 of Zn electrode, 300 mL fluorochemical wastewater, 3 mA cm^{-2} of applied current density.

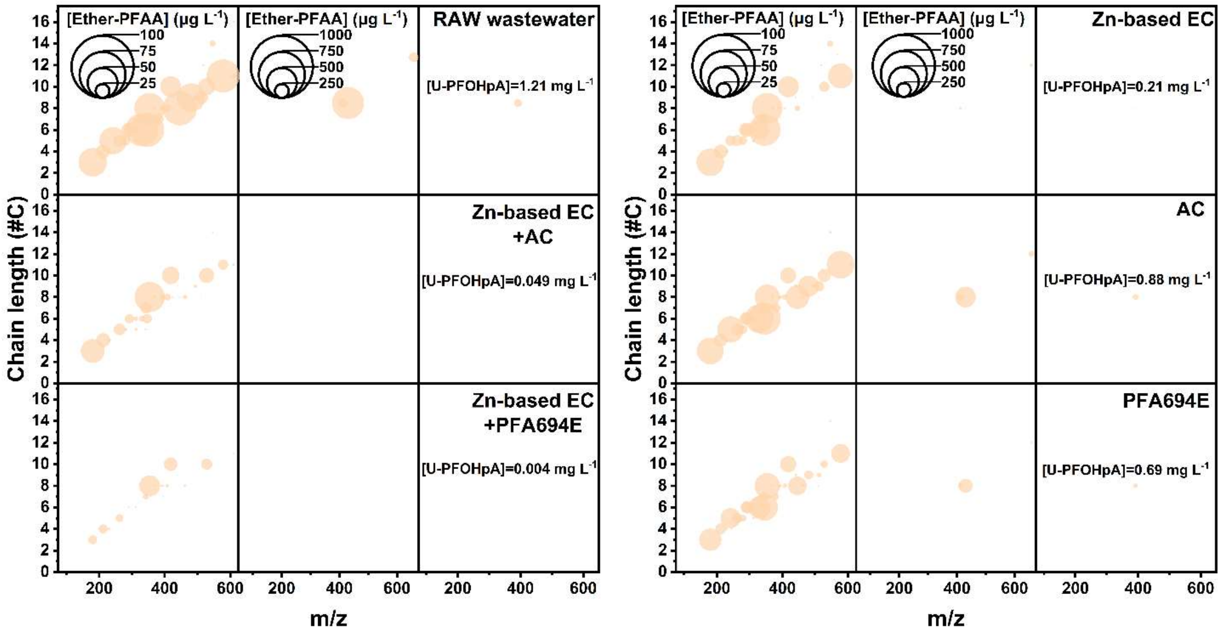

Supplementary Fig. 6. Performance of different systems for the removal of 32 H-PFAA from fluorochemical wastewater. Experiment conditions: 100 cm^2 of Zn electrode, 300 mL fluorochemical wastewater, 3 mA cm^{-2} of applied current density.

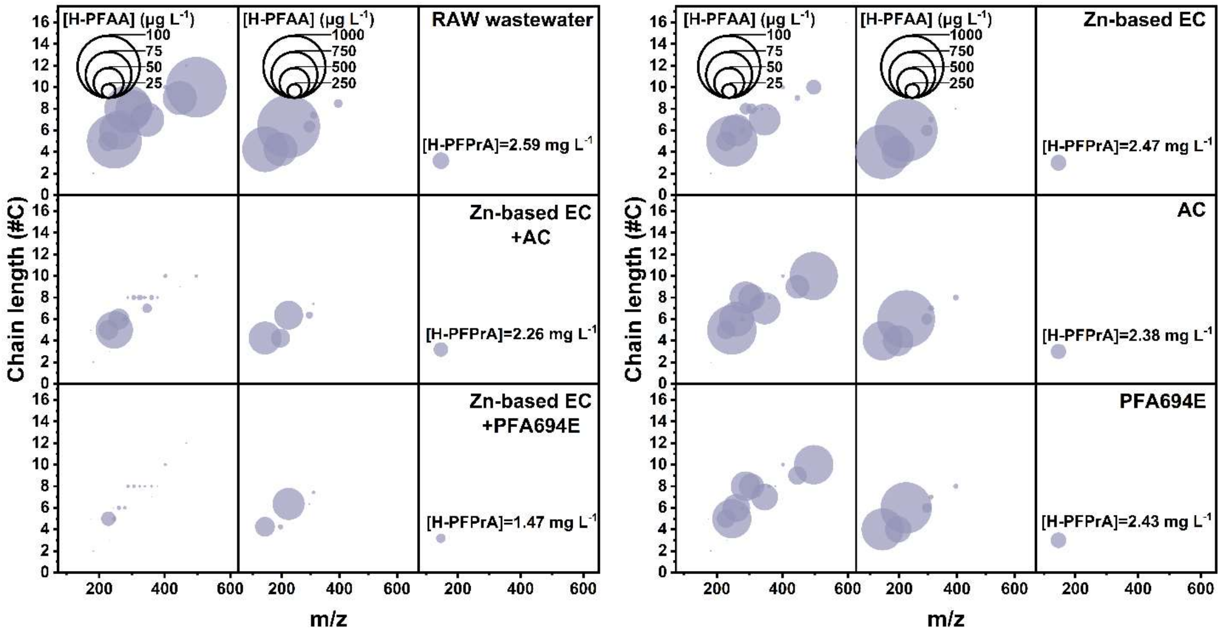

Supplementary Fig. 7. Removal of anions and TOC from fluorochemical wastewater. a, b, c Changes in the concentration of anions and TOC *vs*. time using PFA694E (**a**), AC (**b**) and Zn-based EC (**c**). Experiment conditions: 100 cm^2 of Zn electrode, 300 mL fluorochemical wastewater, 3 mA cm^{-2} of applied current density. All the error bars in this figure represent the standard deviation of the data from duplicate tests.

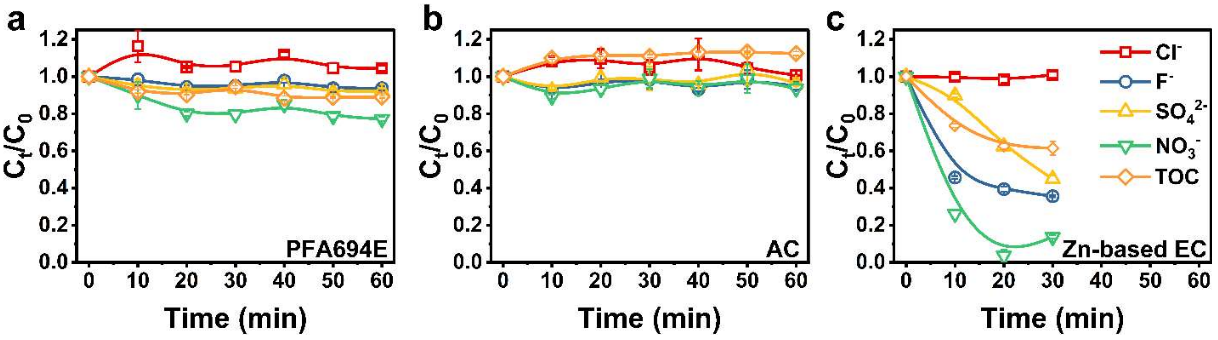

Supplementary Fig. 8 XRD characterization of the zinc hydroxide flocs. The zinc hydroxide flocs in-situ by Zn-based EC in PFAS simulated water under following conditions: initial PFAS concentration of 1.2mM, 1 mA cm^{-2} of applied current density, 20 mM NaCl of electrolyte concentration The zinc hydroxide flocs were freeze-dried before characterizations.

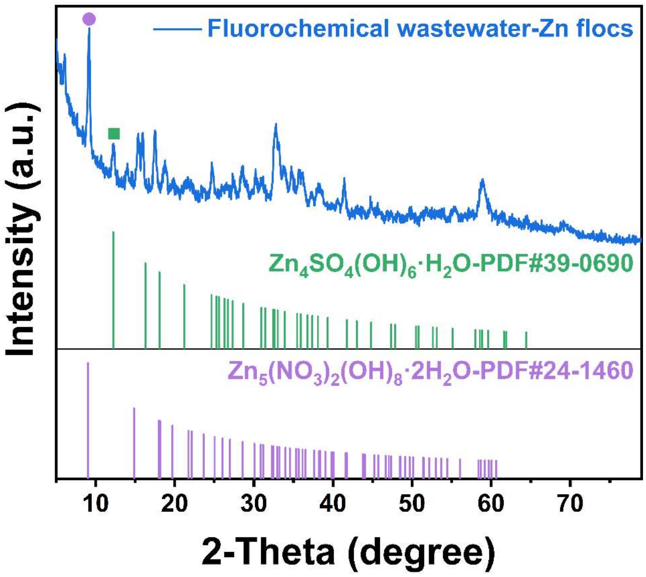

Supplementary Fig. 9. Sorption kinetics of 8 PFAS by Zn-based EC in simulated waters. a, b Changes in the concentration of PFAS (**a**) and adsorbed amount (q_t) of PFAS (**b**) *vs.* time. Experiment conditions: 100 cm^2 of Zn electrode, 300 mL 1.2 mM PFAS simulated waters, 1 mA cm^{-2} of applied current density, 20 mM NaCl of electrolyte concentration, pH=7. All the error bars in this figure represent the standard deviation of the data from duplicate tests.

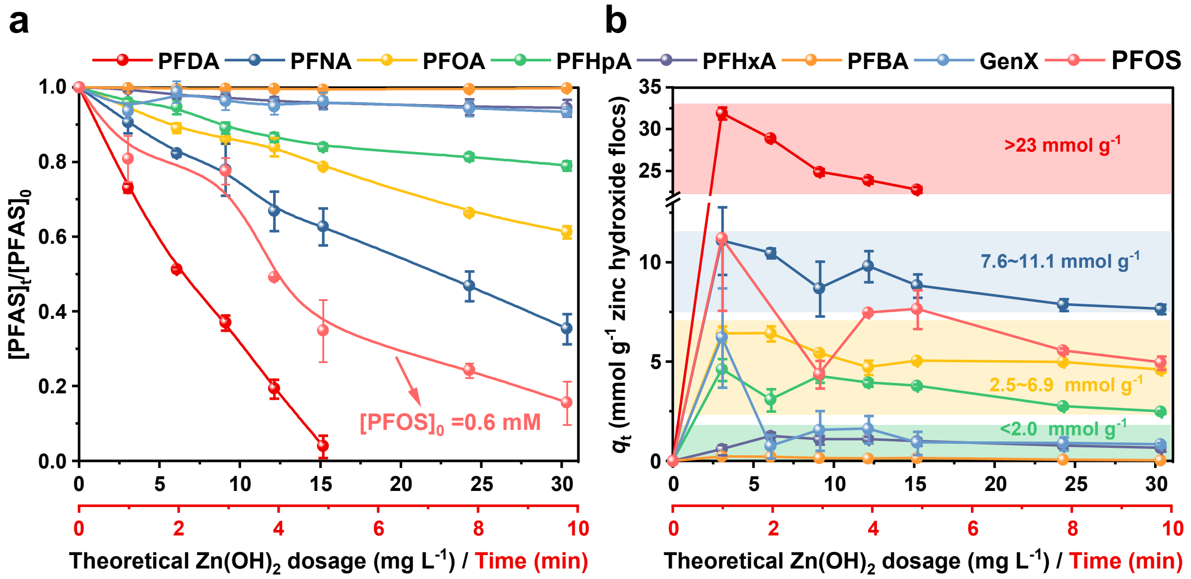

Supplementary Fig. 10 XPS and SEM-EDX characterization of the fresh and PFAS-adsorbed zinc hydroxide flocs. a The XPS spectra. **b** High resolution of C1s XPS spectra. **c** SEM (left) & EDX (right) characterizations. Inset in **c** is the elements mapping of Zn (yellow) and F (green). Experiment conditions for Zn-based EC: 100 cm^2 of Zn electrode, 300 mL simulated solution with or without1.2 mM PFOA, 20 mM NaCl as supporting electrolyte, 1 mA cm^{-2} of applied current density, pH=7. The collected zinc hydroxide flocs were freeze-dried before characterizations.

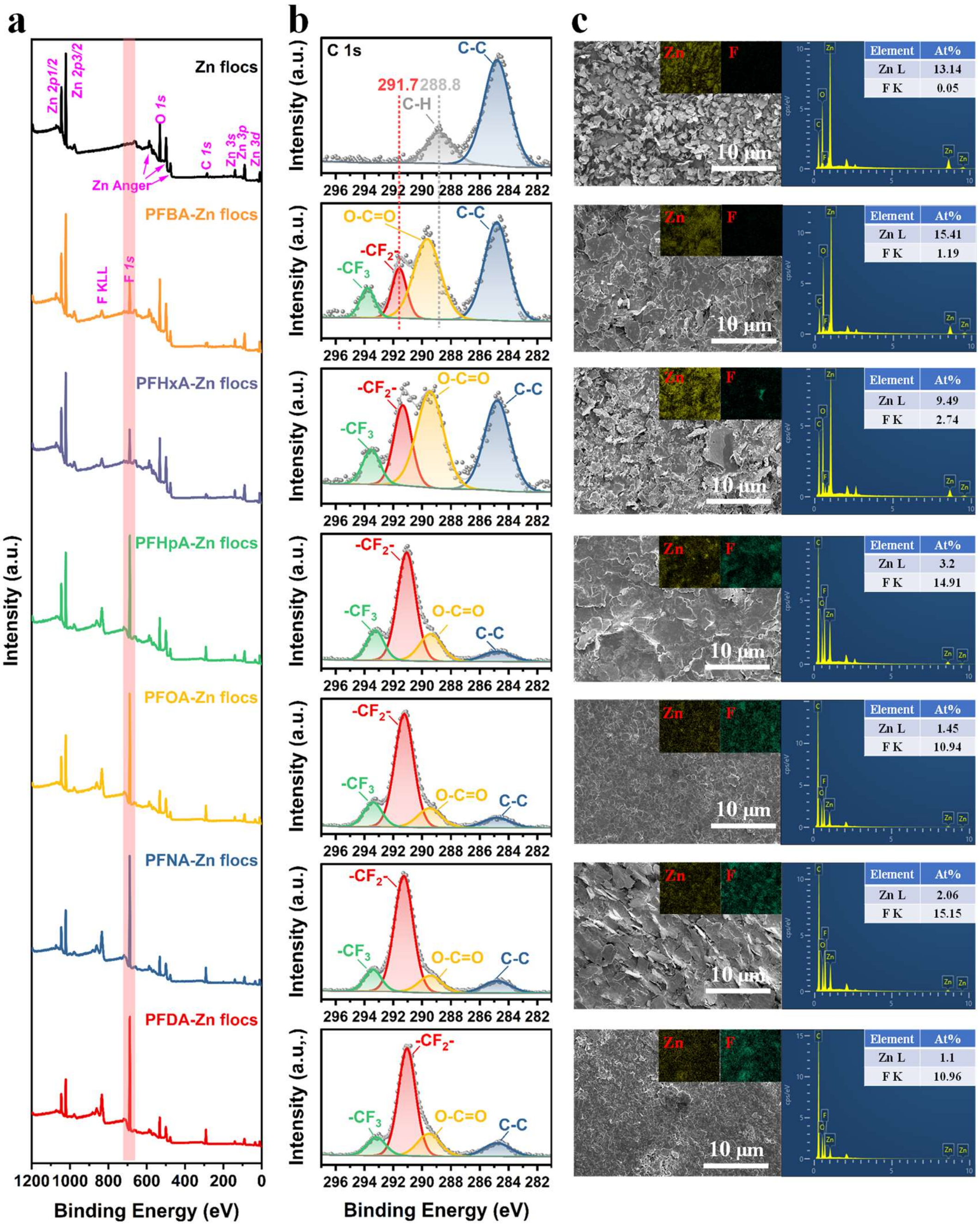

Supplementary Fig. 11. The contact angles of different flocs. Experiment conditions for the formed Zn, Al and Fe flocs by electrocoagulation: current density of 1.0 mA cm^{-2}, electrolyte concentration of 20 mM NaCl, pH=7.

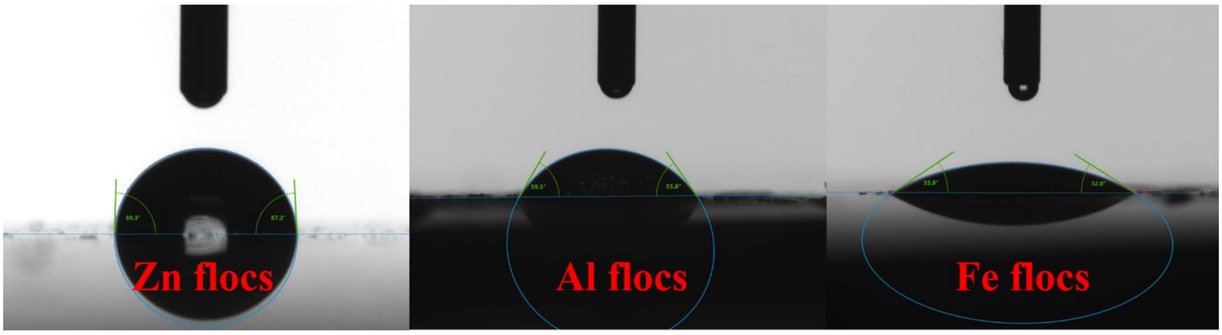

Supplementary Fig. 12. The molecular size of PFDA and PFOA. The molecular size optimized by the Gaussian 09.

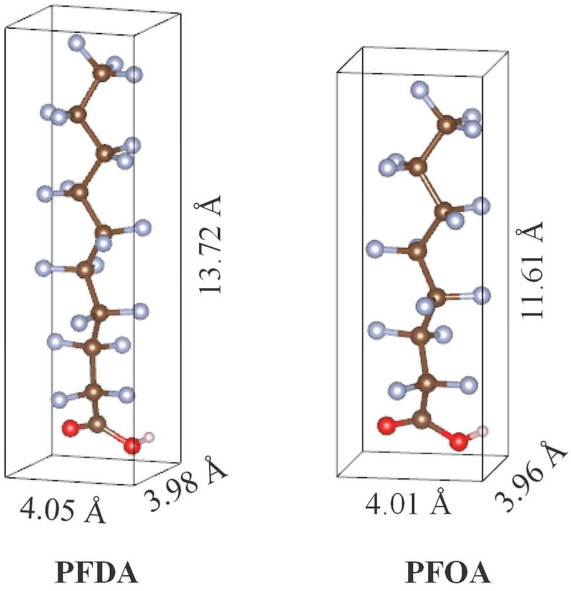

Supplementary Fig. 13 N_2 adsorption-desorption isotherms of zinc hydroxide flocs. Experiment conditions for the formed zinc hydroxide flocs by Zn-based EC: 100 cm^2 of Zn electrode, 300 mL 20 mM NaCl current solution, 1 mA cm^{-2} of applied current density, pH=7. The zinc hydroxide flocs were freeze-dried before characterizations.

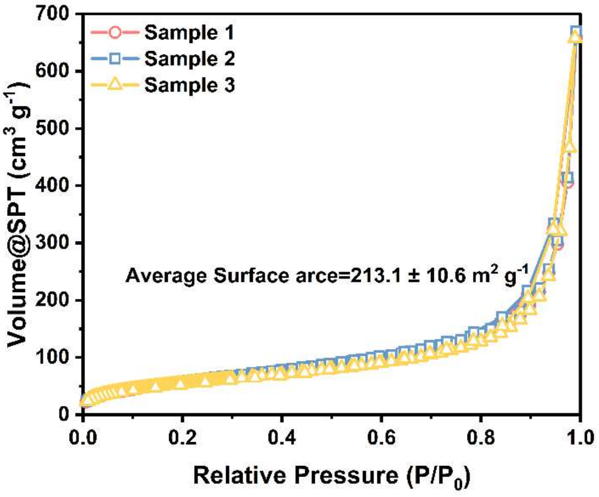

Supplementary Fig. 14. Zeta potentials on the zinc hydroxide flocs *vs.* electrolysis time. 100 cm^2 of Zn electrode, 300 mL PFOA solution (25 mg L^{-1}), 1 mA cm^{-2} of applied current density, 20 mM NaCl as supporting electrolyte. All the error bars in this figure represent the standard deviation of the data from duplicate tests.

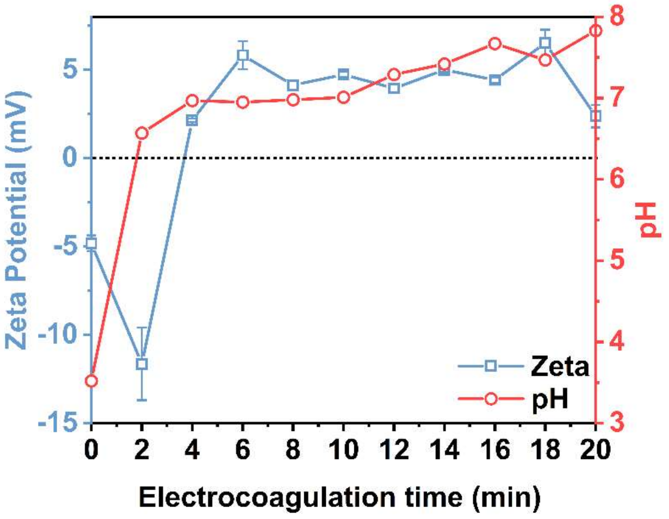

Supplementary Fig. 15. Electrooxidation degradation kinetics of PFAS in the fluorochemical wastewater. a, b, c, d, e Changes in the concentration of C4 PFAS (**a**), C5 PFAS (**b**), C7 PFAS(**c**), C8 PFAS (**d**), I-PFAS (**e**) *vs.* time. Experiment conditions: Flow-through Ti_4O_7 reactive electrochemical membrane system, circulating test mode, 20 mA cm^{-2}.

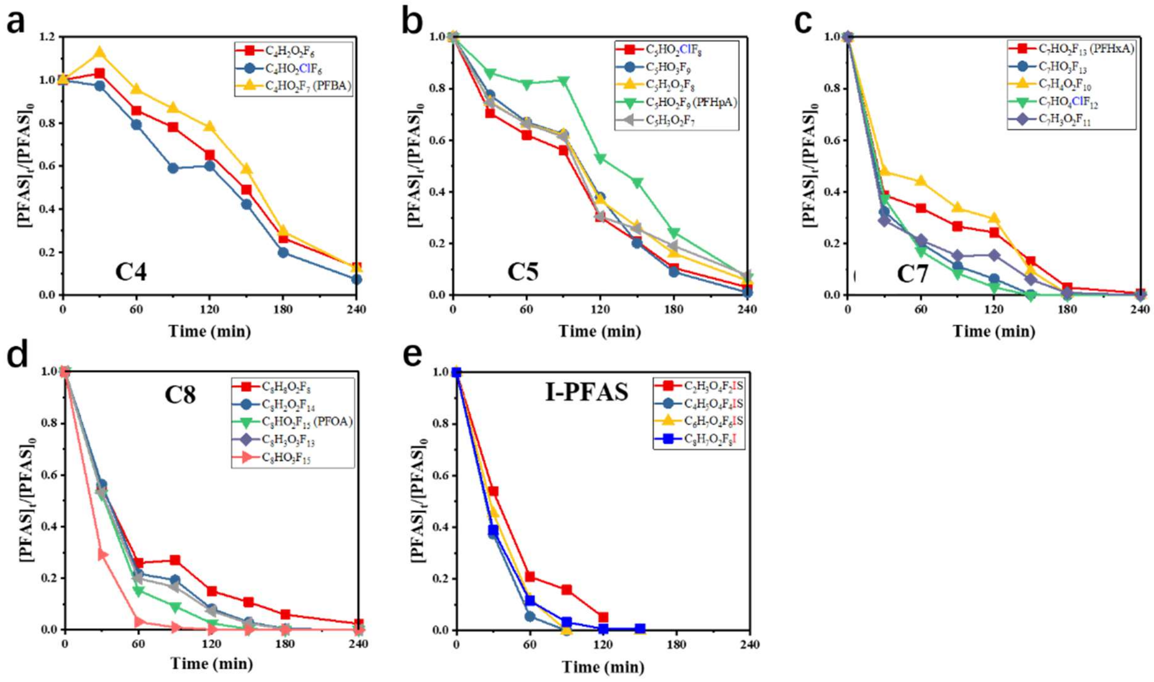

Supplementary Fig. 16. The images of the 20 L pilot Zn-based EC reactor. a, b Front view (**a**) and vertical view (**b**) of the pilot Zn-based EC reactor.

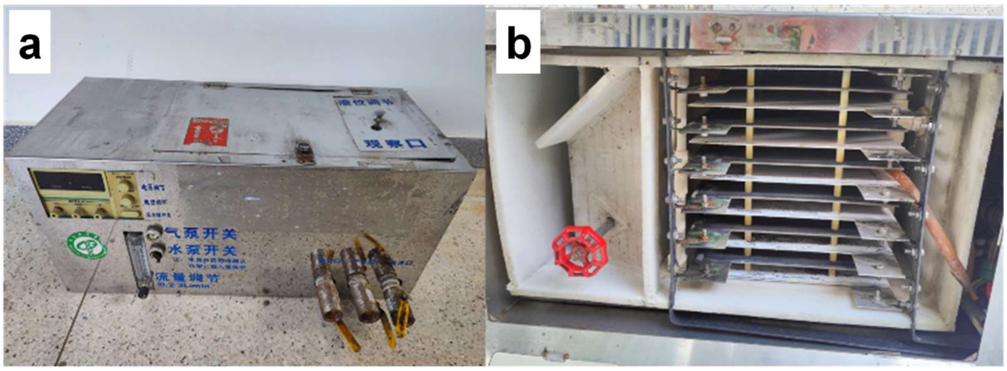

Supplementary Fig. 17. Concentrations of 107 PFAS in the RSSCT influent at 1.03×10^3 BV. **a, b, c, d** Concentration of 9 PFCA (**a**), 14 Cl/I-PFAA (**b**), 52 Ether-PFAA (**c**), and 32 H-PFAA(**d**) in the RSSCT influent at 1.03×10^3 BV. RSSCT of PFA694E adsorption bed fed by untreated or Zn-based EC treated fluorochemical wastewater.

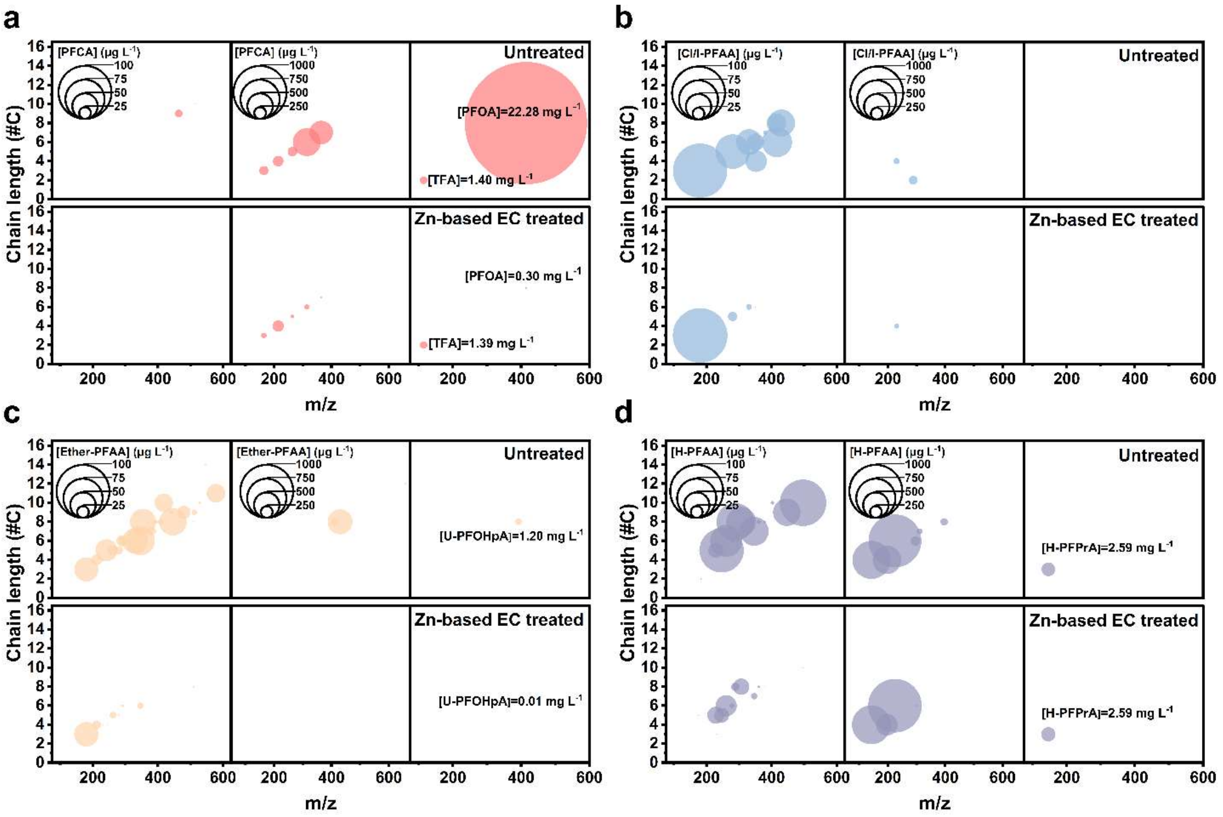

Supplementary Fig. 18. RSSCT breakthrough curves of 23 PFAS. RSSCT breakthrough of PFA694E adsorption bed fed by untreated (**a**) or Zn-based EC treated (**b**) fluorochemical wastewater.

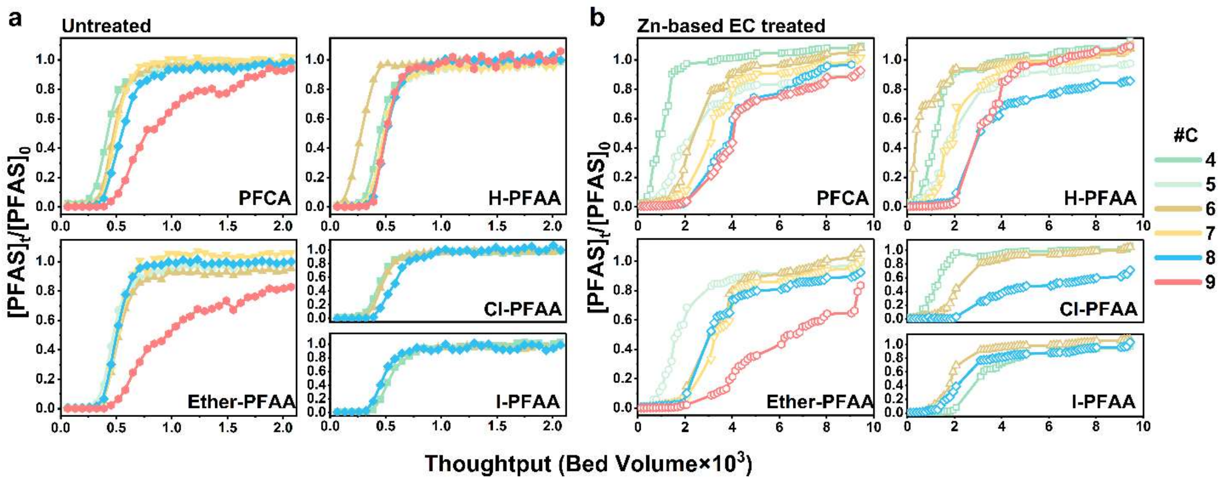

Supplementary Fig. 19. BV$_{80}$ values of 23 PFAS. RSSCT breakthrough of PFA694E adsorption bed fed by untreated or Zn-based EC treated fluorochemical wastewater.

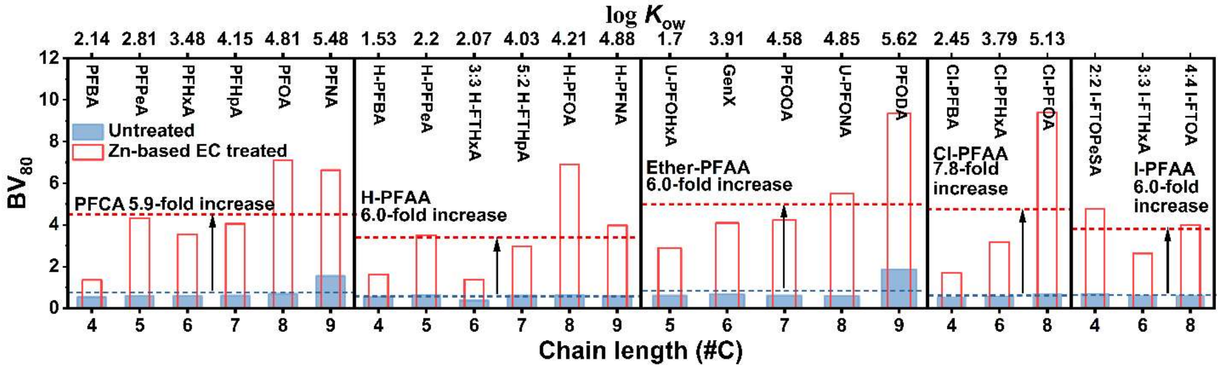

Supplementary Fig. 20. Finite loading of 23 representative PFAS onto the PFA694E bed. RSSCT breakthrough of PFA694E adsorption bed fed by untreated or Zn-based EC treated fluorochemical wastewater. All the error bars in this figure represent the standard deviation of the data from duplicate tests.

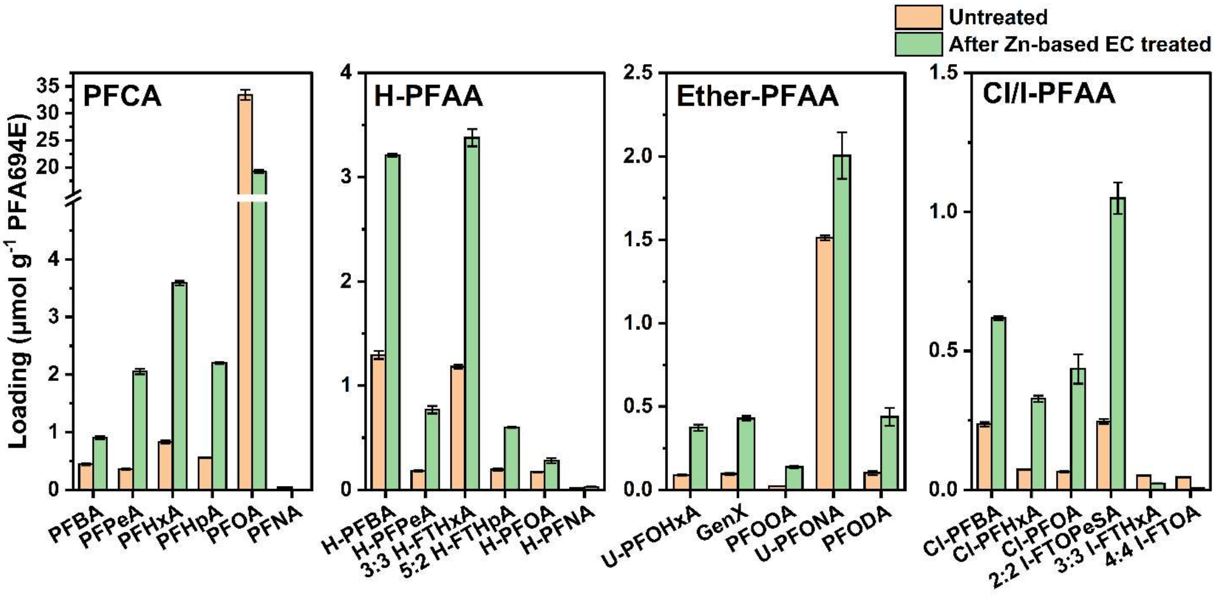

Supplementary Fig. 21. Residue zinc in the effluent of the Zn-based EC and RSSCT column. The drinking water limits of zinc set by the World Health Organization (WHO) is 3 mg L^{-1}. All the error bars in this figure represent the standard deviation of the data from duplicate tests.

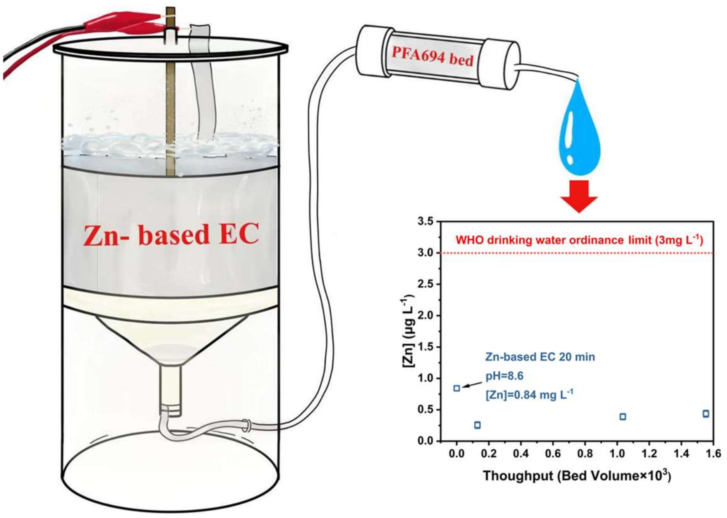

Supplementary References

1. Yu, Q., Zhang, R., Deng, S., Huang, J. & Yu, G. Sorption of perfluorooctane sulfonate and perfluorooctanoate on activated carbons and resin: Kinetic and isotherm study. *Water Res.* **43**, 1150-1158 (2009).

2. Li, J., Li, Q., Li, L. & Xu, L. Removal of perfluorooctanoic acid from water with economical mesoporous melamine-formaldehyde resin microsphere. *Chem. Eng. J.* **320**, 501-509 (2017).

3. Du, Z. et al. Removal of perfluorinated carboxylates from washing wastewater of perfluorooctanesulfonyl fluoride using activated carbons and resins. *J. Hazard Mater* **286**, 136-143 (2015).

4. Zaggia, A., Conte, L., Falletti, L., Fant, M. & Chiorboli, A. Use of strong anion exchange resins for the removal of perfluoroalkylated substances from contaminated drinking water in batch and continuous pilot plants. *Water Res.* **91**, 137-146 (2016).

5. Yang, Y., Zheng, Z., Ji, W., Xu, J. & Zhang, X. Insights to perfluorooctanoic acid adsorption micro-mechanism over Fe-based metal organic frameworks: Combining computational calculation with response surface methodology. *J. Hazard Mater* **395**, 122686 (2020).

6. Mohd Azmi, L. H., Williams, D. R. & Ladewig, B. P. Polymer-assisted modification of metal-organic framework MIL-96 (Al): influence of HPAM concentration on particle size, crystal morphology and removal of harmful environmental pollutant PFOA. *Chemosphere* **262**, 128072 (2021).

7. Sini, K., Bourgeois, D., Idouhar, M., Carboni, M. & Meyer, D. Metal–organic framework sorbents for the removal of perfluorinated compounds in an aqueous environment. *New J. Chem.* **42**, 17889-17894 (2018).

8. Liu, K., Zhang, S., Hu, X., Zhang, K., Roy, A. & Yu, G. Understanding the adsorption of PFOA on MIL-101(Cr)-based anionic-exchange metal–organic frameworks: comparing DFT calculations with aqueous sorption experiments. *Environ. Sci. Technol.* **49**, 8657-8665 (2015).

9. Jun, B. et al. Removal of selected endocrine-disrupting compounds using Al-based metal organic framework: Performance and mechanism of competitive adsorption. *J. Ind. Eng. Chem.* **79**, 345-352 (2019).

10. Clark, C. A., Heck, K. N., Powell. C. D. & Wong, M. S. Highly defective UiO-66 materials for the adsorptive removal of perfluorooctanesulfonate. *ACS Sustainable Chem. Eng.* **7**, 6619-6628 (2019).

11. Li, C. et al. Surface-associated metal catalyst enhances the sorption of perfluorooctanoic acid to multi-walled carbon nanotubes. *J. Colloid Interface Sci.* **377**, 342-346 (2012).

12. Li, Z., Wang, L., Li, Y., Feng, Y. & Feng, W. Carbon-based functional nanomaterials: Preparation, properties and applications. *Compos. Sci. Technol.* **179**, 10-40 (2019).

13. Xiao, L., Ling, Y., Alsbaiee, A., Li, C., Helbling, D. E. & Dichtel. W. R. Beta-cyclodextrin polymer network sequesters perfluorooctanoic acid at environmentally relevant concentrations. *J. Am. Chem. Soc.* **139**, 7689-7692 (2017).

14. Lin, H., Wang, Y., Niu, J., Yue, Z. & Huang, Q. Efficient sorption and removal of perfluoroalkyl acids (PFAAs) from aqueous solution by metal hydroxides generated in situ by electrocoagulation. *Environ. Sci. Technol.* **49**, 10562-10569 (2015).

15. Zhang, D., Luo, Q., Gao, B., Chiang, S. Y. D., Woodward, D. & Huang, Q. Sorption of perfluorooctanoic acid, perfluorooctane sulfonate and perfluoroheptanoic acid on granular activated carbon. *Chemosphere* **144**, 2336-2342 (2016).

16. Ochoa-Herrera, V. & Sierra-Alvarez, R. Removal of perfluorinated surfactants by sorption onto granular activated carbon, zeolite and sludge. *Chemosphere* **72**, 1588-1593 (2008).

17. Zhang, D., He, Q., Wang, M., Zhang, W. & Liang, Y. Sorption of perfluoroalkylated substances (PFASs) onto granular activated carbon and biochar. *Environ. Technol.* **42**, 1798-1809 (2021).

18. Shahrokhi, R., Hubbe, M. A. & Park, J. Comparative assessment of activated carbon and anion exchange resin for short- and long-chain per- and poly-fluoroalkyl substances sorption: Insight into performance and mechanism. *J. Water Process Eng.* **64**, 105703 (2024).

19. Fang, Y. *et al.* Removal of per- and polyfluoroalkyl substances (PFASs) in aqueous film-forming foam (AFFF) using ion-exchange and nonionic resins. *Environ. Sci. Technol.* **55**, 5001-5011 (2021).

20. Li, R. et al. Efficient removal of per- and polyfluoroalkyl substances from water with zirconium-based metal–organic frameworks. *Chem. Mater.* **33**, 3276-3285 (2021).

21. Niu, Z., Wang, Y., Lin, H., Jin, F., Li, Y. & Niu, J. Electrochemically enhanced removal of perfluorinated compounds (PFCs) from aqueous solution by CNTs-graphene composite electrode. *Chem. Eng. J.* **328**, 228-235 (2017).

22. Li, X., Chen, S., Quan, X. & Zhang, Y. Enhanced adsorption of PFOA and PFOS on multiwalled carbon nanotubes under electrochemical assistance. *Environ. Sci. Technol.* **45**, 8498-8505 (2011).

23. Ching, C., Lin, Z., Dichtel, W. R. & Helbling, D. E. Evaluating the performance of novel cyclodextrin polymer granules to remove perfluoroalkyl acids (PFAAs) from Water. *ACS ES&T Eng.* **3**, 661-670 (2023).

24. Wang, J., Lin, Z., Dichtel, W. R. & Helbling, D. E. Perfluoroalkyl acid adsorption by styrenic β-cyclodextrin polymers, anion-exchange resins, and activated carbon is inhibited by matrix constituents in different ways. *Water Res.* **260**, 121897 (2024).

25. Yang, A., Ching, C., Easler, M., Helbling, D. E. & Dichtel, W. R. Cyclodextrin polymers with nitrogen-containing tripodal crosslinkers for efficient PFAS adsorption. *ACS Mater. Lett.* **2**, 1240-1245 (2020).

26. Wang, W. et al. Adsorption behavior and mechanism of emerging perfluoro-2-propoxypropanoic acid (GenX) on activated carbons and resins. *Chem. Eng. J.* **364**, 132-138 (2019).

27. Guo, H. et al. Roles of varying carbon chains and functional groups of legacy and emerging per-/polyfluoroalkyl substances in adsorption on metal-organic framework: Insights into mechanism and adsorption prediction. *Environ Res.* **251**, 118679 (2024).

28. Tan, H., Pan, C., Yin, C. & Yu, K. Toward systematic understanding of adsorptive removal of legacy and emerging per-and polyfluoroalkyl substances (PFASs) by various activated carbons (ACs). *Environ Res.* **233**, 116495 (2023).
